# Interplay between gut microbiota and tryptophan metabolism in type 2 diabetic mice treated with metformin

**DOI:** 10.1128/spectrum.00291-24

**Published:** 2024-08-20

**Authors:** Yvhao Xie, Xinxin Li, Qingshi Meng, Jinjun Li, Xin Wang, Liying Zhu, Weiwei Wang, Xiaoqiong Li

**Affiliations:** 1 College of Animal Science, Shanxi Agricultural University, Taigu, China; 2 State Key Laboratory for Managing Biotic and Chemical Threats to the Quality and Safety of Agro-products & Food Sciences Institute, Zhejiang Academy of Agricultural Sciences, Hangzhou, China; 3 State Key Laboratory of Animal Nutrition, Institute of Animal Sciences, Chinese Academy of Agricultural Sciences, Beijing, China; Institute of Microbiology, Chinese Academy of Sciences, Beijing, China

**Keywords:** tryptophan metabolism, gut microbiota, type 2 diabetes mellitus, metformin, LC-MS/MS, indole-3-lactic acid, indole-3-propionic acid

## Abstract

Tryptophan (TRP) metabolites have been identified as potent biomarkers for complications of type 2 diabetes mellitus (T2DM). However, it remains unclear whether the therapeutic effect of metformin in T2DM is related to the modulation of TRP metabolic pathway. This study aims to investigate whether metformin affects TRP metabolism in T2DM mice through the gut microbiota. A liquid chromatography-tandem mass spectrometry method was established to determine 16 TRP metabolites in the serum, colon content, urine, and feces of T2DM mice, and the correlations between metabolites and the T2DM mice gut microbiota were performed. The method demonstrated acceptable linearity (*R*^2^ > 0.996), with the limit of quantification ranging from 0.29 to 69.444 nmol/L for 16 analytes, and the limit of detection ranging from 0.087 to 20.833 nmol/L. In T2DM mice, metformin treatment effectively restored levels of indole-3-lactic acid (ILA), indole-3-propionic acid (IPA), and the ILA/IPA ratio, along with several aryl hydrocarbon receptor ligands in the serum, with a notable impact in the colon but not in the urine. This restoration was accompanied by a shift in the relative abundance of *Dubosiella*, *Turicibacter*, *RF39*, *Clostridia_UCG-014*, *and Alistipes*. Spearman’s correlation analysis revealed positive correlations between *Turicibacter* and *Alistipes* with IPA and indole-3-acetic acid. Conversely, these genera displayed negative correlations with ILA and kynurenine. In addition, our study revealed the presence of endogenous indole pathway in germ-free mice, and the impact of metformin on endogenous TRP metabolism in T2DM mice cannot be disregarded. Further research is needed to investigate the regulation of TRP metabolism by metformin.

**IMPORTANCE:**

This study provides valuable insights into the interrelationship between metformin administration, changes in the tryptophan (TRP) metabolome, and gut microbiota in type 2 diabetes mellitus (T2DM) mice. Indole-3-lactic acid (ILA)/indole-3-propionic acid (IPA) emerges as a potential biomarker for the development of T2DM and prediction of therapeutic response. While the indole metabolic pathway has long been associated exclusively with the gut microbiome, recent research has demonstrated the ability of host interleukin-4-induced-1 to metabolize TRP. The detection of indole derivatives in the serum of germ-free mice suggests the existence of inherent endogenous indole metabolic pathways. These findings deepen our understanding of metformin's efficacy in correcting TRP metabolic disorders and provide valuable directions for further investigation. Moreover, this knowledge may pave the way for the development of targeted treatment strategies for T2DM, focusing on the gut microbiome and restoration of associated TRP metabolism.

## INTRODUCTION

Type 2 diabetes mellitus (T2DM) and its complications incur significant economic costs on healthcare systems and national economies, resulting in an increasing global medical burden (1). Metformin, a synthetic biguanide, currently serves as the primary treatment option for T2DM patients, particularly those with obesity who do not respond to diet control or physical exercise alone (2). It enhances insulin sensitivity, suppresses hepatic glucose production, reduces intestinal glucose absorption, and promotes glucose uptake and utilization. Furthermore, Metformin supports weight management, lowers lipid levels, and helps prevent certain vascular complications (3).

The regulatory effect of metformin on glucose metabolism is influenced by the crosstalk between the host and gut microbiota (4, 5). Short-chain fatty acids (SCFAs), secondary bile acids, and tryptophan (TRP) metabolites are three currently most studied categories of metabolites involved in host-microbiota interactions (6–8). A common signature of gut microbiome alterations in patients with T2DM is a reduction in butyrate-producing taxa, such as Clostridiales (9). Metformin restores the abundance of SCFA-producing bacteria, such as *Blautia*, *Faecalibacterium* spp., resulting in increased fecal concentrations of butyrate and propionate in patients with T2DM and obesity. This restoration process contributes to improvements in glucose tolerance and fasting blood sugar levels (4, 10, 11). Furthermore, metformin modulates the bile acid pool by reducing the abundance of *Bacteroides fragilis* and its bile salt hydrolase activity, thereby regulating glucose tolerance and metabolic disorders (12). Additionally, emerging evidence from animal and cohort studies suggests that TRP metabolism can also impact metabolic health (6, 13). However, it remains unclear whether the pathways involved in TRP metabolism are also implicated in the response of metformin monotherapy to glucose metabolic homeostasis.

TRP, an essential aromatic amino acid, is closely linked to the risk of diabetes (6, 13). An increased ratio of serum kynurenine (KYN) to TRP is correlated with metabolic syndrome and obesity (6, 14). The serum levels of both KYN and the KYN/TRP ratio are significantly elevated in diabetic patients (14). Recent cohort studies have a positive association between indole-3-lactic acid (ILA) and T2DM risk (13), whereas indole-3-propionic acid (IPA) has been found to be positively correlated with increased insulin levels (15, 16) and negatively correlated with T2DM (13). Reduced production of aryl hydrocarbon receptor (AHR) ligands by gut microflora is a crucial factor in the pathogenesis of metabolic syndromes such as obesity and diabetes. These ligands include the TRP metabolites such as indole-3-acrylic acid (IA), indole (Ind), IPA, indole-3-acetic acid (IAA), and indole-3-aldehyde (IAld) (17). The loss of the AHR’s protective effects in promoting repair, inhibiting inflammation, and maintaining intestinal homeostasis contributes to this pathogenesis. Metformin restores the levels of AHR ligands, namely IA, IPA, Ind, and IAA in the liver of mice induced with saccharin/sucralose (17). Hence, we hypothesized that balancing the profile of TRP metabolites could enhance the efficacy of metformin treatment, particularly in T2DM.

Liquid chromatography-tandem mass spectrometry (LC-MS/MS) is a highly sensitive and specific method that enables a comprehensive assessment of biomarkers during the onset and development of diseases such as T2DM (18). This technique has been employed to detect the content of TRP and its metabolites in various biological tissues (19–22). LC-MS/MS has successfully measured the levels of various compounds including TRP, L-KYN, 5-hydroxytryptamine (5-HT), and l-glutamic acid in serum and brain tissue (23). Several rapid and accurate LC-MS/MS methods have been reported for determining TRP metabolites as biomarkers in diseases such as depression (23) and glioblastoma (24).

To the best of our knowledge, no targeted assay has been developed or applied for the detection of TRP and its metabolites profiles as biomarkers in T2DM, and this knowledge gap served as the basis for the objective of our study. We have developed a rapid and sensitive assay using LC-MS/MS to simultaneously determine 16 TRP metabolites in mouse serum, feces, intestinal segments, and urine samples. The method can be utilized to explore changes in TRP metabolites in T2DM mice induced by a high-fat and high-sugar diet combined with streptozotocin. Additionally, the correlation between TRP metabolism and the gut microbiota was examined.

## RESULTS

### LC-MS/MS method development and validation

**Fig 1:**
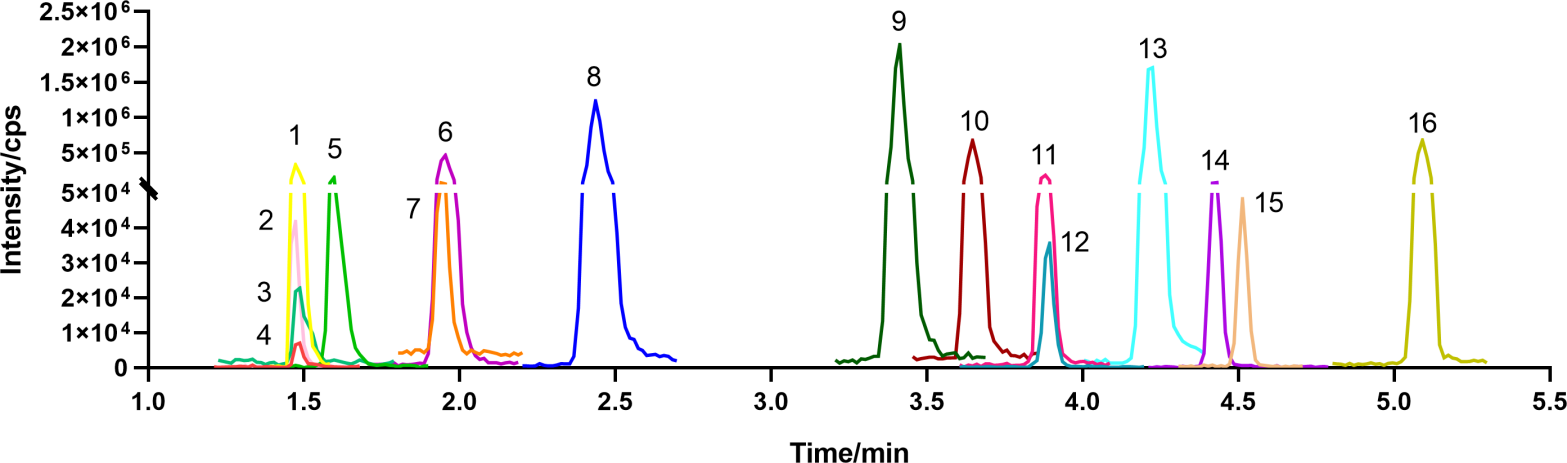
MRM chromatogram of 16 metabolites optimized by LC-MS/MS. List of compounds: 1: 5-hydroxytryptamine (5-HT); 2: Indole-3-acetaldehyde (IAAld); 3: Indole-3-acetic acid (IAA); 4: Indole-3-pyruvic acid (IPyA); 5: Kynurenine (KYN); 6: L-Tryptophan (TRP); 7: Indole-3-acrylic acid (IA); 8: Tryptamine (TrA); 9: Indole-3-acetamide (IAM); 10: 2-Oxindole (2-Ox); 11: Indole-3-lactic acid (ILA); 12: Indole-3-aldehyde (IAld); 13: Indole (Ind); 14: 3-Skatole (3MI); 15: Tryptophol (IEt); and 16: Indole-3-propionic acid (IPA).

Chromatographic parameters were established using gradient inversion, and the appropriate analytical peaks were obtained. The chromatograms of the 16 compounds in positive ion mode are shown in Fig. 1; Fig. S1. No interference peaks of any of the analytes or the isotope-labeled internal standard (ISTD) were observed under the selected conditions in the serum and fecal samples, and there was no interference between the analytes and the ISTD in the blank water.

Standard solutions of different concentrations were mixed with the ISTD and analyzed by LC-MS/MS. The linear relationship between the analyte area ratio (analyte area/ISTD area) and its nominal concentration was analyzed. Each standard curve consisted of a minimum of five points with different concentrations to ensure accurate quantification. The method exhibited good selectivity. Table 1 summarizes the calibration curve, coefficient of determination, the limit of quantitation (LOQ), the limit of detection (LOD), and the intra- and inter-day precision of this determination. The correlation coefficients for the calibration curves of each compound were >0.99, achieving a linear range with *R*^2^ > 0.996. The LOQ (signal-to-noise ratio, S/*N* = 10) for the 16 analytes ranged from 0.29 to 69.444 nmol/L, while the LOD (S/*N* = 3) ranged from 0.087 to 20.833 nmol/L. The relative standard deviation (RSD, %) values of inter- and intra-day repeatability and precision were below 20%, except for indole-3-pyruvic acid (IPyA) in blood samples and 3-skatole (3-methylindole, 3MI) in stool samples. The analyte recovery rate was evaluated by adding indole-3-acetic-2,2-d2 as the ISTD during the initial sample extraction, with retention time and mass in Table 2. The recovery rates in serum and feces were 88.63% and 94.54%, respectively (Table 1).

**TABLE 1:** Calibration curves, LODs, LOQs, intraday accuracy, precision, and extraction recovery of the analyte of interest in the sample^*d*^

Compound	Abbrev	Calibration curve	*R* ^2^	LOD^*a*^ (nM)	LOQ^*b*^ (nM)	Rrecision (RSD, %)			
						Intra-day		Inter-day	
						Blood	Faeces	Blood	Faeces
Tryptophol	IEt	*y* = 6 × 10^6^*x* + 58,084	0.9999	0.427	1.425	−^d^	4.08	−	4.49
Indole-3-pyruvic acid	IPyA	*y* = 243,349*x* − 10,079	0.9966	20.833	69.444	1.88	3.59	21.15	5.95
Indole-3-acrylic acid	IA	*y* = 7 × 10^6^*x* − 10,802	0.9986	0.985	3.283	3.07	4.03	2.27	2.96
5-hydroxytryptamine	5-HT	*y* = 1 × 10^6^*x* − 33,891	0.9992	2.804	9.346	14.90	3.36	11.55	10.33
Kynurenine	KYN	*y* = 1 × 10^6^*x* + 4444	0.9995	0.666	2.219	14.56	1.95	13.72	8.08
Indole-3-acetic acid	IAA	*y* = 4 × 10^6^*x* + 63,647	0.9994	0.334	1.114	15.36	4.38	8.88	4.25
Indole-3-aldehyde	IAld	*y* = 3 × 10^7^*x* + 482,624	0.9996	0.087	0.290	−	4.76	−	4.96
Tryptamine	TrA	*y* = 8 × 10^6^*x* + 173,898	0.9991	0.101	0.335	−	6.24	−	10.43
3-Skatole	3MI	*y* = 337,627*x* + 874.92	0.9981	11.710	39.063	13.02	0.47	7.64	25.34
Tryptophan	TRP	*y* = 3 × 10^6^*x* + 20,678	0.9988	0.479	1.595	8.04	5.74	12.52	4.96
Indole-3-lactic acid	ILA	*y* = 2 × 10^6^*x* + 6026.8	0.9996	0.356	1.188	3.20	4.97	13.00	6.89
Indole-3-propionic acid	IPA	*y* = 6 × 10^6^*x* + 83,546	0.9996	0.246	0.818	4.14	4.96	12.93	5.99
Indole	Ind	*y* = 9 × 10^6^*x* + 175,017	0.9988	0.259	0.862	−	4.97	−	4.98
Indole-3-acetaldehyde	IAAld	*y* = 578,808*x* + 1269.5	0.9988	1.482	4.941	2.84	4.97	8.41	5.58
Indole-3-acetamide	IAM	*y* = 2 × 10^7^*x* + 808,586	0.9983	0.134	0.448	−	−	−	−
2-Oxindole	2-Ox	*y* = 3 × 10^6^*x* + 29,160	0.9990	1.169	3.897	−	4.24	−	6.62
Indole-3-acetic-2,2-d2	IAA-2, 2-d_2_^*c*^	*y* = 2 × 10^6^*x* + 31,079	0.9975	1.053	3.511	1.70	3.16	3.26	3.56

^*a*^ Limit of detection.

^*b*^ Limit of quantification.

^*c*^ The recovery rate of IAA-2, 2-d2 in serum and feces was 88.63% and 94.54%, respectively.

^*d*^ "−", Below lower limit of quantification.

**TABLE 2:** MRM transition and mass spectrometry parameters optimized for each analyte and ISD in positive ionization mode

Compound	Abbrev	Formula	Retention time (min)	Q1 mass (Da)	Q3 mass (Da)	Time (ms)	Declustering potential (V)	Collision energy (V)	Collision cell exit potential (V)
5-hydroxytryptamine	5-HT	C_10_H_12_N_2_O	1.36	177.00	115.00	20.00	56.00	37.00	14.00
Kynurenine	KYN	C_10_H_12_N_2_O_3_	1.61	209.09	192.10	20.00	90.00	15.00	15.00
Tryptophan	TRP	C_11_H_12_N_2_O_2_	1.92	205.00	115.00	20.00	26.00	53.00	12.00
Tryptamine	TrA	C_10_H_12_N_2_	2.39	161.09	144.09	20.00	60.00	17.00	10.00
Indole-3-acetamide	IAM	C_10_H_10_N_2_O	3.36	175.20	130.20	20.00	70.00	27.00	10.00
Indole	Ind	C_8_H_7_N	3.39	118.00	91.00	20.00	100.00	30.00	11.00
Indole-3-pyruvic acid	IPyA	C_11_H_9_NO_3_	3.71	204.20	158.30	20.00	40.00	15.00	13.00
Indole-3-acetic-2,2-d2	IAA-2,2-d_2_	C_10_H_11_NO_2_	3.84	178.02	132.10	20.00	76.00	23.00	13.00
Indole-3-lactic acid	ILA	C_11_H_11_NO_3_	3.96	206.00	130.00	20.00	70.00	30.00	14.00
Indole-3-acetaldehyde	IAAld	C_10_H_9_NO	3.96	160.20	118.10	20.00	76.00	32.00	10.00
Indole-3-aldehyde	IAld	C_9_H_7_NO	4.40	146.03	118.04	20.00	60.00	21.00	14.00
Indole-3-acetic acid	IAA	C_10_H_9_NO_2_	4.71	176.02	130.03	20.00	86.00	25.00	10.05
2-Oxindole	2-Ox	C_8_H_7_NO	4.71	134.05	105.00	20.00	50.00	29.00	10.00
Tryptophol	IEt	C_10_H_11_NO	4.82	162.00	130.10	20.00	75.00	45.00	13.00
Indole-3-acrylic acid	IA	C_11_H_9_NO_2_	5.26	188.00	115.00	20.00	61.00	39.00	12.00
Indole-3-propionic acid	IPA	C_11_H_11_NO_2_	5.66	190.00	77.00	20.00	51.00	21.00	10.00
3-Skatole	3MI	C_9_H_9_N	8.23	132.02	105.00	20.00	60.00	30.00	14.00

### Metformin-modified TRP metabolism in serum, colonic content, and urine samples of T2DM mice

**Fig 2:**
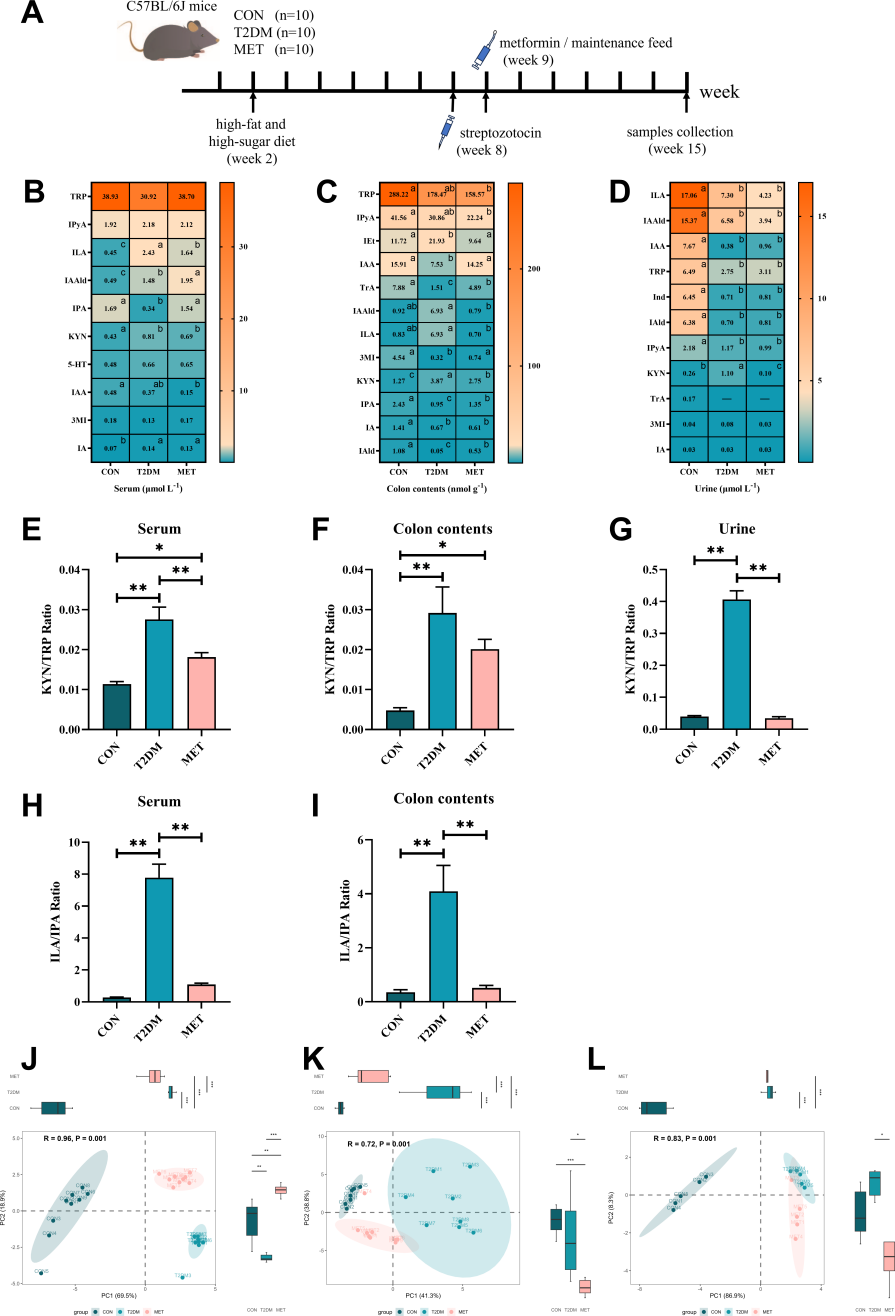
T2DM and metformin treatment modified mice TRP metabolism. Modeling and metformin intervention method of T2DM mice (**A**). The concentration of TRP and its metabolites in serum (B, *n* = 8–9), colon contents (C, *n* = 7–9), and urine (D, *n* = 5) samples were determined by LC-MS/MS. The ratio of KYN to TRP was calculated in serum (**E**), colon contents (**F**), and urine (**G**) samples. The ratio of ILA to IPA was calculated in serum (**H**) and colon contents (**I**) samples. Principal component analysis of the metabolites in serum (**J**), colon contents (**K**), and urine (**L**) samples based on Bray-Curtis distance, ANOSIM was used to calculate *R* and *P* values, the box plot illustrates the distribution of principal component values for each group. The heatmap displays the mean values, with red indicating higher values and blue indicating lower values. The data in the histograms are presented as the mean ± standard error of the mean (SEM). Statistical differences in the heatmaps and histograms were analyzed using one-way ANOVA with *post hoc* comparisons using Bonferroni. In the heatmap, different letters indicate significant differences (*P*  <  0.05), and significant differences in the histograms are denoted by (*) for *P* < 0.05 and (**) for *P* < 0.01.

Metformin partially rectified the concentration of TRP metabolites in the serum, colon contents, and urine of T2DM mice (Fig. 2A). Figure 2A illustrates the procedure for establishing a T2DM mouse model and metformin intervention. As displayed in Fig. 2B, the concentrations of ILA, indole-3-acetaldehyde (IAAld), and KYN were significantly elevated (*P* < 0.05) in the serum samples of the T2DM group compared to the CON group, while the concentrations of IPA and IA were significantly reduced (*P* < 0.05). Treatment with metformin effectively decreased ILA levels while significantly boosting IPA levels back to normal levels in T2DM mice.

**Fig 3:**
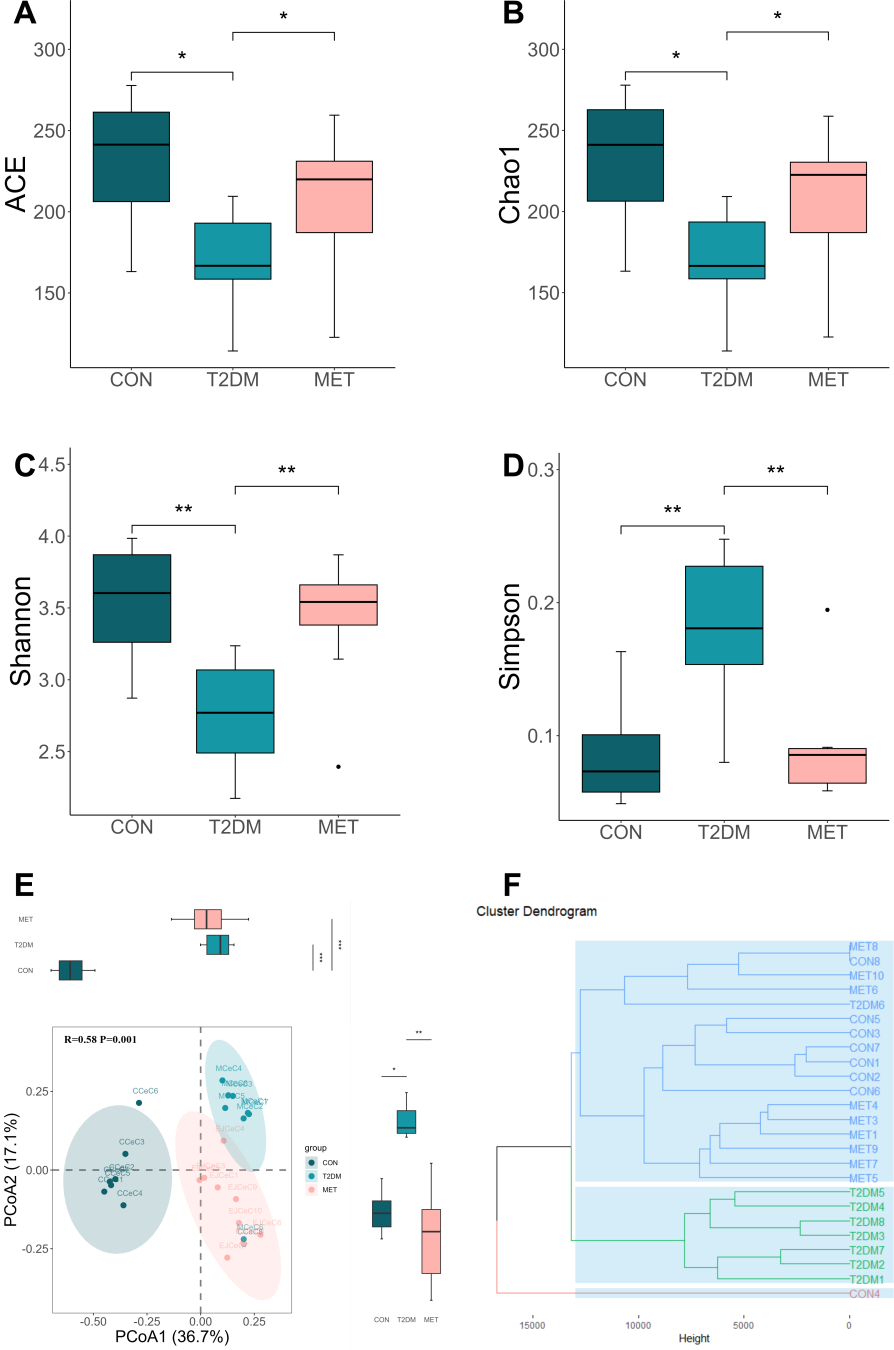
Alpha and beta diversity of Colon flora in different groups of mice. Colon contents from three groups of mice (*n* = 8–9) were collected for 16S rRNA Sequencing. Alpha diversity is expressed as mean ± SEM and analyzed using Kruskal-Wallis with *post hoc* comparisons using Bonferroni, including ACE index (**A**), Chao1 index (**B**), Shannon index (**C**), and Simpson index (**D**). Principal coordinate analysis (**E**) of the colon microbial community composition of the CON, T2DM, and MET mice based on Bray-Curtis distance. Hierarchical clustering utilizing the Bray-Curtis distance (**F**). ANOSIM was used to calculate *R* and *P* values (**F**). Statistical significance is denoted by (*) for *P* < 0.05 and (**) for *P* < 0.01.

In the colon contents (Fig. 3C), the concentrations of tryptophol (IEt) and KYN were significantly higher (*P* < 0.05) in the T2DM group in comparison to the healthy controls, while the levels of IAA, tryptamine (TrA), 3MI, IPA, IA, and IAld were significantly lower (*P* < 0.05) than those observed in the CON group. Additionally, although the observed trend did not reach statistical significance, there was a tendency toward an increase in IAAld (*P* = 0.0535) and ILA (*P* = 0.0628) in the T2DM group. After metformin treatment, the concentrations of IAA, TrA, 3MI, IPA, and IAld in colon contents significantly increased, accompanied by a significant reduction in the levels of IEt, IAAld, ILA, and KYN. These findings suggest that metformin intervention effectively mitigates dysregulated TRP metabolism within the gut microbiota.

Furthermore, metformin treatment significantly reduced the increased KYN concentration in the urine samples of T2DM mice, but did not lead to significant improvements in the decreased levels of ILA, IAAld, IAA, TRP, Ind, IAld, and IPyA (Fig. 2D). Notably, metformin treatment did not result in any significant alterations in TRP metabolism in fecal samples of T2DM mice (Fig. S2A).

In addition, the KYN/TRP ratio in the serum, colon contents, and urine samples of the T2DM group was significantly increased (*P* < 0.01). While metformin therapy restored normal KYN/TRP ratios in the serum and urine (Fig. 2E and G), it did not fully normalize the KYN/TRP ratios in colon contents (Fig. 2F). The KYN/TRP ratio is frequently used to assess the activity of the extrahepatic Trp-degrading enzyme indoleamine 2,3 dioxygenase (IDO). Although KYN and TRP values varied, the increase of KYN/TRP ratio in T2DM indicated an increase in host KYN pathway metabolism (dominated by IDO) within the TRP metabolic pathways (14), while the corresponding microbiota-dominated indole metabolic pathway decreased (6). Treatment with metformin showed benefits in correcting the imbalance between host and bacterial TRP metabolism in T2DM. Interestingly, the ILA/IPA ratio in the serum and colon contents samples was also significantly increased in the T2DM group (*P* < 0.01), and this ratio was restored after metformin treatment (Fig. 2H and I).

Principal component analysis (PCA) was carried out to elucidate the alterations in the TRP metabolite profiles in serum, colonic contents, and urine among the three groups. ANOSIM analysis, employing Bray-Curtis distances, unveiled notable distinctions in the TRP metabolite profiles found in serum (*R* = 0.96, *P* < 0.01), colon contents (*R* = 0.72, *P* < 0.01), and urine (*R* = 0.83, *P* < 0.01) among the CON, T2DM, and MET groups (Fig. 2J, K and L; Fig.S2C). Remarkably, the position of the confidence ellipse in the MET group fell between that of the CON and MET groups, suggesting a partial restoration of the TRP metabolic profile post-metformin treatment.

Partial least squares discriminant analysis (PLS-DA) was conducted to analyze metabolites from both serum samples and colon content samples (Fig. S3A and C). We employed PLS-DA variable importance projection scores to assess the significance of various TRP metabolites in the context of metformin treatment for T2DM. Notably, IPA, IAAld, and ILA emerged as crucial variables among the serum metabolites (Fig. S3B). Conversely, IAA, 3MI, and IPA were identified as key variables in the colon contents (Fig. S3D). These findings highlight the potential of these metabolites as biomarkers for predicting the onset and progression of T2DM.

### Metformin treatment recovered the alpha diversity and partially restored the gut microbiota composition in T2DM mice

The rarefaction curve for the analysis of alpha diversity using amplicon sequence variants (ASVs) in different groups confirms the adequate sequencing depth of each sample (Fig. S4). The ACE, Chao1, Shannon, and Simpson indices (Fig. 3A through D) demonstrated a significant decrease in the richness and diversity of the gut microbiota in the T2DM group compared to the CON group. However, metformin treatment notably restored these indices.

Principal coordinates analysis was conducted at the amplicon ASV level to assess the distinct patterns of bacterial community structures within the three different groups (Fig. 3E). ANOSIM analysis, utilizing Bray-Curtis distances, unveiled significant distinctions among the CON, T2DM, and MET groups (*R* = 0.58, *P* < 0.01). The hierarchical clustering based on Bray-Curtis distance indicated that the microbial composition of the MET group exhibited higher similarity to the CON group compared to the samples from the T2DM group (Fig. 3F).

**Fig 4:**
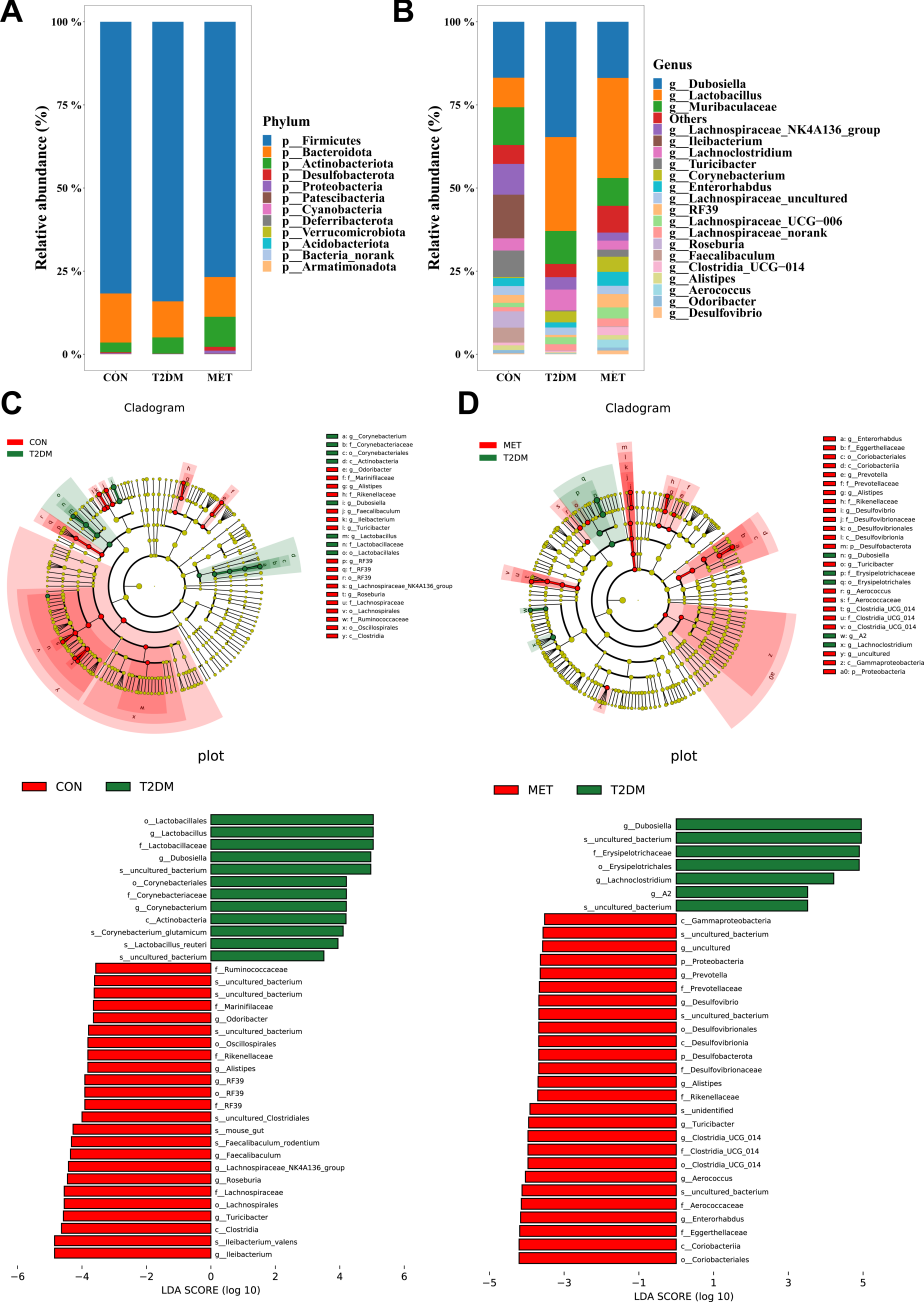
T2DM and metformin treatment modified the composition of intestinal flora in different groups (*n* = 8–9) of mice. Through the annotation of 16S rDNA sequencing results using the SILVA database. The stacked bar graph illustrated the predominant phyla (**A**) and genera (**B**) with abundance exceeding 1%. Linear discriminant analysis effect size (LEfSe) was performed to determine significant differences in gut microbiota between different groups. Cladogram for significant differences of taxonomic representations between CON and T2DM groups (**C**), or T2DM and MET groups (**D**), with colored nodes from the inner circle to the outer circle representing taxa from gate to genus. Linear discriminant analysis (LDA) score for taxa differing between CON and T2DM groups (**C**), or T2DM and MET groups (**D**) with LDA score threshold >3.5.

Based on the annotated 16S rDNA sequencing results from the SILVA database, Fig. 4A and B illustrated the phyla and genera respectively, with abundances surpassing 1%. To identify differentially abundant taxa among the groups, we employed linear discriminant analysis effect size (LEfSe). Figure 4C and D demonstrate that the T2DM group exhibited lower abundance of SCFA-producing bacteria, including *Faecalibaculum*, *Lachnospiraceae NK4A136 group*, *Alistipes*, *Roseburia*, and *Turicibacter*. Notably, after metformin intervention, the abundance of *Allisteria* was restored, while *Faecalibaculum* and *Turicibacter* experienced partial restoration.

### Effect of metformin on TRP metabolism partially mediated by specific bacterial taxa

**Fig 5:**
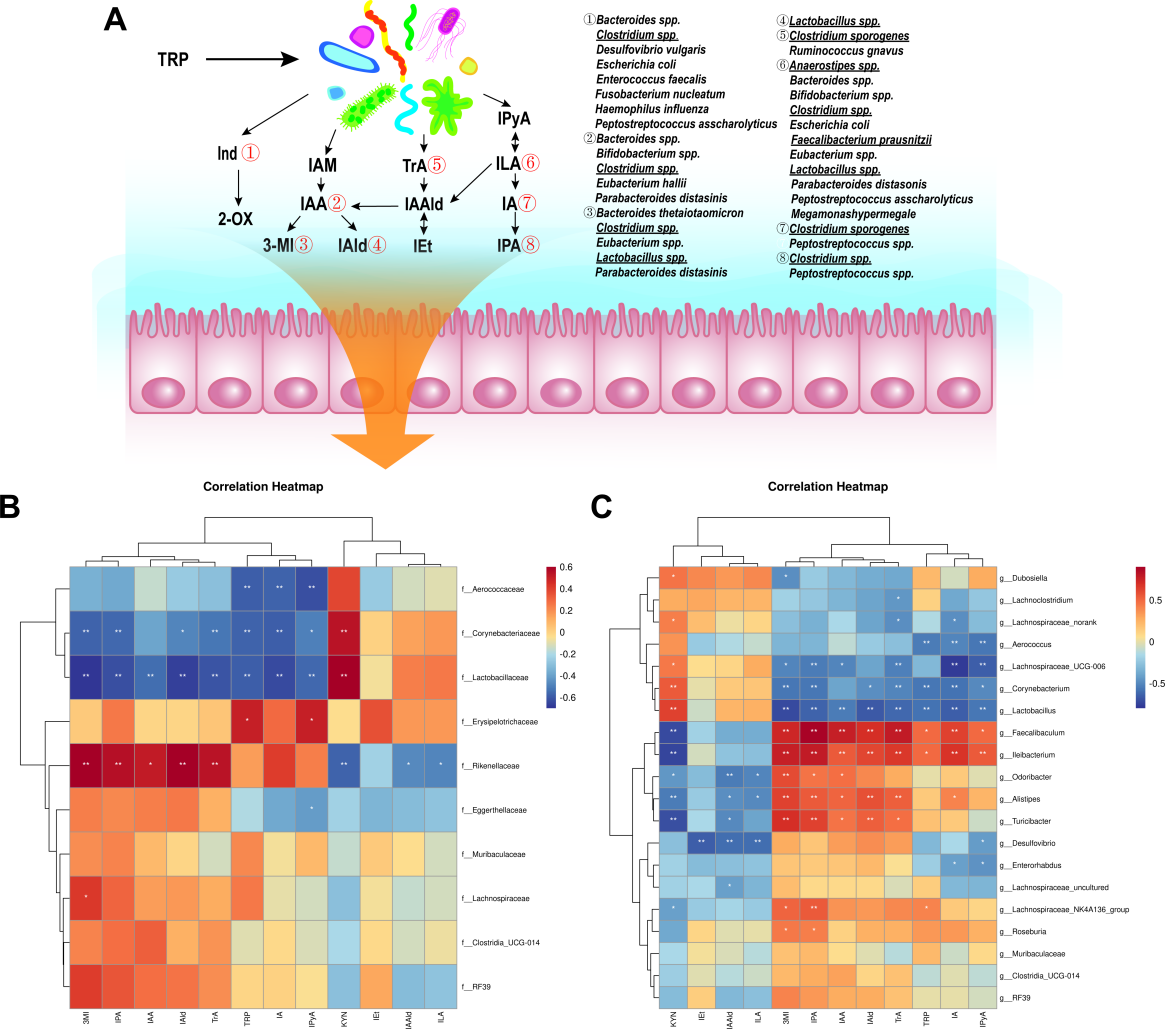
Correlation between the prominent genera and TRP metabolites. (**A**) The illustration of the TRP metabolic pathway (25, 26). The heatmaps displaying the Spearman correlation between colonic microbiota at the (**B**) family level and (**C**) genus level with TRP metabolites. The intensity of the colors represents the degree of association, with red indicating a positive correlation and blue indicating a negative correlation. Significant correlations are denoted by (*) for *P* < 0.05 and (**) for *P* < 0.01.

Utilizing the PICRUST2 method to predict the functional profile of the sequencing results, we observed a significant enrichment of TRP metabolism-related pathways following metformin treatment (Fig. S5). To better understand the intricate interplay between the gut microbiota and microbial-derived indole derivatives (Fig. 5A), we conducted Spearman correlation analysis to examine the association between TRP metabolism and the abundance of gut microbiota. As shown in Fig. 5B and C, we found a positive correlation between *Lactobacillaceae*, *Lactotobacillus*, *and Dubosiella* (which were enriched in the T2DM group) with KYN, while displaying a negative correlation with 3MI and IPA. Additionally, we observed positive correlations between *Faecalibaculum*, *Turicibacter*, and *Alistipes* (which decreased in the T2DM group and were restored by metformin treatment) with IPA, IAA, IAld, TrA, and 3MI. Conversely, these genera displayed negative correlations with ILA, KYN, and IAAld.

### Germ-free mice possess an endogenous indole pyruvate pathway

**Fig 6:**
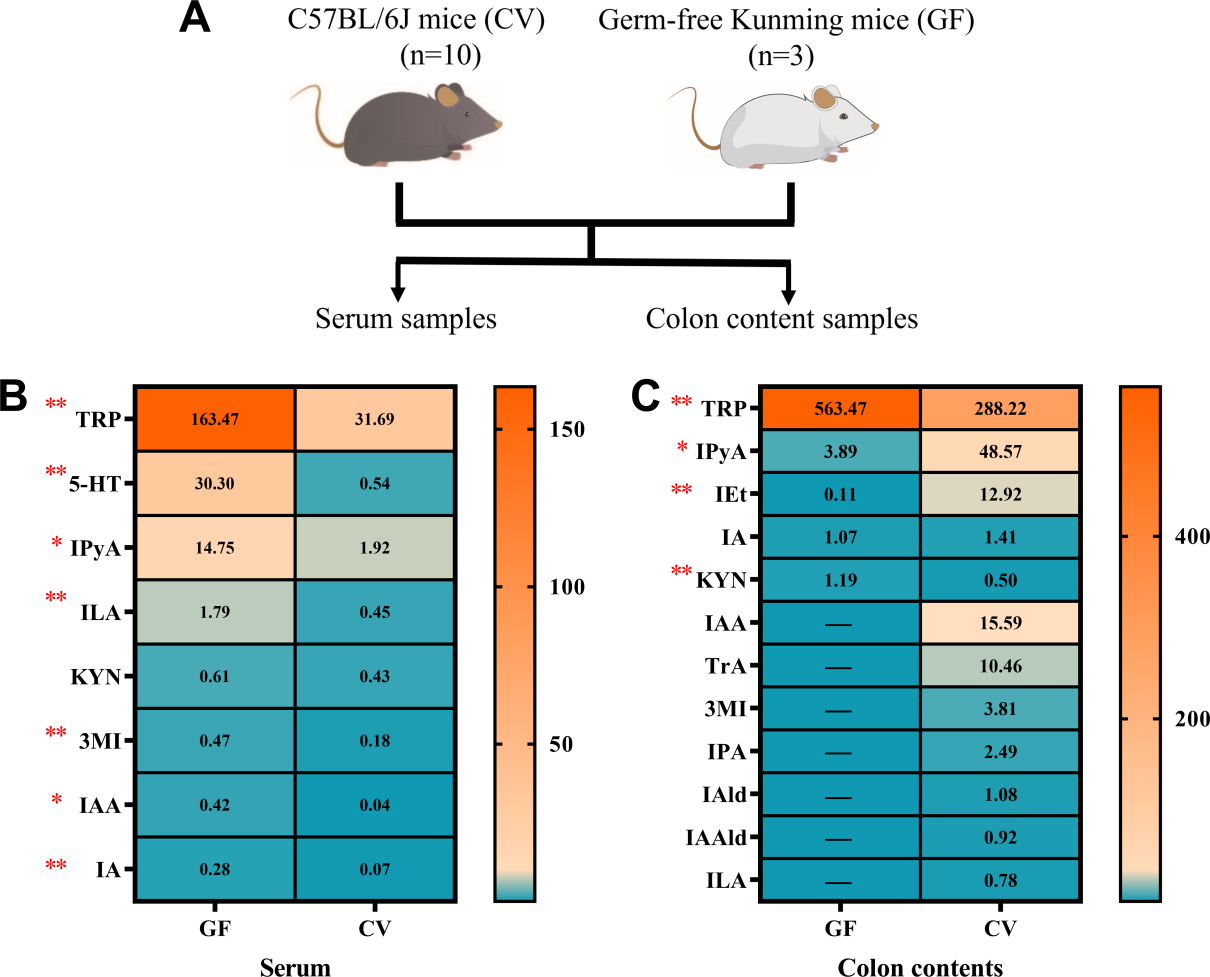
The comparison of TRP metabolite concentrations between GF and CV mice. The origin of the samples (**A**). The content of TRP and its metabolite in serum (**B**) or colon contents samples (**C**) between GF (*n* = 3) and CV mice (*n* = 10) were performed using the LC-MS/MS platform. The heatmap illustrates the mean values, with redder colors indicating higher values and bluer colors indicating lower values. Statistical differences were assessed using an independent-sample *t* test, with significance denoted at *P* < 0.05 (*), *P* < 0.01 (**).

To investigate the exclusive origins of indole derivatives from the gut microbiota, we analyzed the levels of TRP metabolites in serum and colonic content from germ-free (GF) and conventional (CV) mice (Fig. 6A). Surprisingly, we successfully detected TRP, 5-HT, IPyA, ILA, KYN, 3MI, IAA, and IA in the serum of both GF and CV mice (Fig. 6B). However, CV mice exclusively exhibited the presence of IPA and IAAld. In the colonic content, only five compounds (TRP, IPyA, IEt, IA, and KYN) were detected in the colonic content of GF mice (Fig. 6C), while the concentrations of IAA, TrA, 3MI, IPA, IAld, IAld, and ILA in CV mice also exceeded the detection limit.

## DISCUSSION

The gut microbiota plays a direct or indirect role in controlling four main TRP metabolic pathways, which produce 5-HT, KYN, TrA, and other indole derivatives (6, 27), due to this involvement, TRP metabolism holds significant potential as predictive biomarkers for diabetes risk and as therapeutic targets for T2DM. Several TRP metabolites, such as IPA, IPyA, IAAld, IAld, IAA, Ind, and 2-oxindole (2-OX), have been identified to exert functions like anti-inflammatory, immunosuppressive, and intestinal mucosal protective through the AHR (21, 28, 29). Certain microbial genera, such as *Subdoligranulum*, *Lactobacillus*, and *Paraprevotella*, have been shown to correlate with TRP degradation (30). In our study, we established a LC-MS/MS method to quantify TRP and its metabolites in the serum, colonic content, urine, and fecal samples from T2DM mice treated with metformin. Changes in TRP metabolism profiles were analyzed alongside 16S rDNA sequencing to examine the involvement of gut microbiota in TRP metabolism in T2DM mice subjected to metformin treatment.

During the establishment of the LC-MS/MS method, we have made appropriate improvements to the approach originally proposed by Wang et al. (23). Calibration standards, LOD, and LOQ samples were prepared in blank ultrapure water to evaluate linearity and sensitivity, while quality control (QC) samples were prepared using serum and fecal suspensions to evaluate inter-day and intra-day precision. Our methodology successfully detected TRP and its 15 metabolites within a 12-min timeframe. This is faster than the method reported by Sadok et al. (23 min) (31), and comparable to that of Takahashi et al. (32). In contrast to the approach of Hu et al. (33), which involves different steps for different compounds, our method follows a uniform procedure, ensuring simplicity and convenience. Moreover, our approach encompasses a broader range of TRP metabolites, specifically indole derivatives, compared to the methods of Wang et al. (23), Sofie et al. (34), and Pedraz-Petrozzi et al. (35). Consequently, this method enables the investigation of the impact of gut microbiota TRP metabolism on individual health and facilitates the exploration of targeted intervention strategies to improve disease conditions. Moreover, the RSD values were in accordance with the guidelines for validating bioanalytical methods outlined by the US Food and Drug Administration (36). Subsequently, we applied this method to quantify the profiles of TRP metabolites in serum, colon, and urine samples obtained from a mouse model with T2DM that was treated with metformin.

TRP and its metabolite concentrations have been demonstrated to be correlated with T2DM. TRP regulates glucose metabolism and insulin levels in animals with T2DM in a GPR142-dependent manner (37, 38). TRP levels in plasma samples were significantly lower in diabetic subjects compared to non-diabetic subjects (39). The decrease in TRP levels may be attributed to the upregulation of IDO caused by inflammatory factors in patients with T2DM, ultimately resulting in the metabolism of TRP into KYN (40). Consequently, there is a positive correlation between KYN levels and T2DM (41). A previous study demonstrated higher urinary excretion of KYN in patients with T2DM (42). In accordance with this, our study also confirmed elevated KYN levels in the serum, colonic content, and urine samples in the T2DM group. And after metformin treatment, there was a certain degree of decrease in KYN levels in these tissues. The KYN/TRP ratio is an established clinical biomarker for assessing the activity of indoleamine 2,3-dioxygenase (IDO) (43). Patients with T2DM and poor glycemic control had significantly elevated serum KYN/TRP ratios (44). In our study, treatment with metformin resulted in the suppression of elevated KYN/TRP ratio in serum, colonic content, and urine samples of diabetic mice. Although there was no significant difference observed in the colonic content samples, but this may be related to the decrease in TRP concentration caused by the promotion of TRP indole metabolic pathway by the microbial community.

In addition to the restoration of the KYN/TRP ratio, the primary effect of metformin treatment was normalizing the ILA/IPA ratio. Metformin treatment reduced ILA accumulation and alleviated IPA deficiency in mice with T2DM. Our findings aligned with previous research indicating a positive association between ILA and the risk of T2DM (13), while revealing a negative association between IPA and diabetes risk (15, 16). The metabolism of TRP is intricate, involving multiple bacterial strains in the biosynthesis of indole derivatives (25). Currently, various bacteria including *Lactobacillus* spp., *Bifidobacterium* spp., as well as specific species of *Clostridium* and *Bacteroides* species have been identified as producers of ILA. *Lactobacillus spp*. convert TRP to ILA via an indoleacetic acid dehydrogenase (ILDH) (25). However, only a limited number of genera, specifically *Clostridium* and *Peptostreptococcus*, are known to produce IPA (26, 45). And *Clostridia_UCG-014* is considered related to the TRP metabolism. The conversion of ILA to IA and its subsequent conversion to IPA is facilitated by the activity of Acyl-CoA dehydrogenase in IPA-producing bacteria (46). We observed an enrichment in both Lactobacillaceae and *Lactobacillus,* and a decrease in the abundance of *Clostridia_UCG-014* in T2DM mice compared to normal mice. The content of *Lactobacillus* was significantly higher in T2DM patients compared to healthy individuals (5, 47). Although *Lactobacillus* abundance was not reduced with metformin treatment, but there was an increase in *Clostridia_UCG-014* abundance. Therefore, our speculation is that metformin may reduce the production of ILA by inhibiting ILDH rather than by inhibiting *Lactobacillus* abundance. Another plausible explanation is that ILA, serving as a precursor to IPA, is utilized by *Clostridia_UCG-014* in the MET group to facilitate increased production of IPA. Correlation analysis revealed a significant negative correlation between IPA and *Lactobacillus*, while a positive correlation was observed between IPA and *Clostridia_UCG-014*.

The therapeutic effect of metformin on T2DM was also related to other indole derivatives. The colonic content of the T2DM group exhibited significantly lower levels of IAA, TrA, 3MI, and IAld compared to the CON group. After metformin treatment, there was partial or complete recovery observed in the concentrations of these indole derivatives. A previous study has demonstrated a correlation between decreased levels of AhR ligands, such as IA, IPA, Ind, and IAA derived from the colonic microbiota, and metabolic syndrome (17). Furthermore, the IAld-producing *Lactobacilli* contribute to the transcription of AhR-dependent IL-22, thus promoting gut mucosal homeostasis (48). The restoration of IAA levels after metformin treatment may be associated with a notable increase in Actinobacteria, which has been demonstrated to produce IAA (49). Patients with T2DM showed an elevation in Erysipelotrichaceae abundance (50). In line with our findings, T2DM mice subjected to metformin treatment displayed a reduction in Erysipelotrichaceae abundance (51). *Dubosiella*, a member of the Erysipelotrichaceae family, is considered to be inversely associated with obesity and diabetes (52, 53). However, although *Dubosiella* is associated with the host’s inflammatory response and lipid metabolism, there is no conclusive evidence of its direct involvement in TRP metabolism.

Interestingly, in our study, the abundance of SCFA-producing bacteria *Faecalibaculum*, *Lachnospiraceae NK4A136 group*, *Alistipes*, *Roseburia*, and *Turicibacter* was lower in the T2DM group. After administration of metformin, the abundance of *Allisteria* was restored, *Faecalibaculum* and *Turicibacter* were partially restored. Previous studies have demonstrated an increase in the abundance of *Alistipes* (54, 55) and *Turicibacter* (56) following metformin treatment. *Turicibacter* is a taxon known for its anti-inflammatory properties influencing host bile acid and lipid metabolism (57, 58) and promoting the intestinal production of 5-HT (59). *Alistipe*s is a bacterium that capable of producing indole via the enzyme tryptophanase, which converts TRP to indole (60, 61). The Spearman correlation analysis revealed significant positive correlations between *Turicibacter* and *Alistipes* with IPA and IAA, while exhibiting a significant negative correlation with KYN and ILA. Furthermore, Research has indicated a positive correlation between the *RF39* and the levels of IPA in the serum of middle-aged women (62). *Enterorhabdus* is considered to be a butyrate producer that degrades amino acids (63). In summary, we had determined that metformin has notable effects on TRP metabolism in mice with T2DM. These effects appeared to be partially mediated by the gut microbiota, particularly by specific bacterial taxa such as *Turicibacter*, *Enterorhabdus*, *RF39*, *Clostridia_UCG-014*, and *Alistipes*, which potentially regulate AHR agonists. Future research that incorporates metagenomic sequencing is needed in order to obtain more precise findings regarding the role of specific gut microbes and the associated functional genes involved in TRP metabolism.

Previous studies and reviews have generally indicated that indole and its derivatives are exclusively produced through the metabolic activity of gut microorganisms on TRP (64–68). However, recent reports have shed light on an additional endogenous pathway involving interleukin-4-induced gene 1, which encodes a protein with l-amino acid activity that preferentially catalyzes the conversion of TRP to indole-3-pyruvic acid and indole derivatives (IAAld, IAA, IAld, and ILA) within host cells (69–71). Our research findings suggest that metformin intervention may regulate TRP metabolism in the gut and body of mice by restoring the abundance of *Turicibacter*, *Enterorhabdus*, *RF39*, *Clostridia_CG-014*, and *Alistipes*. However, we have confirmed the presence of endogenous TRP indole metabolism pathways (ILA, 3MI, and IAA) in GF mice. Therefore, the potential for metformin to regulate TRP metabolism through endogenous indole pathways cannot be dismissed. More research is needed to explore the precise mechanisms by which beneficial indole catabolites can be manipulated to improve gut homeostasis and mitigate the progression of T2DM.

### Conclusions

**Fig 7:**
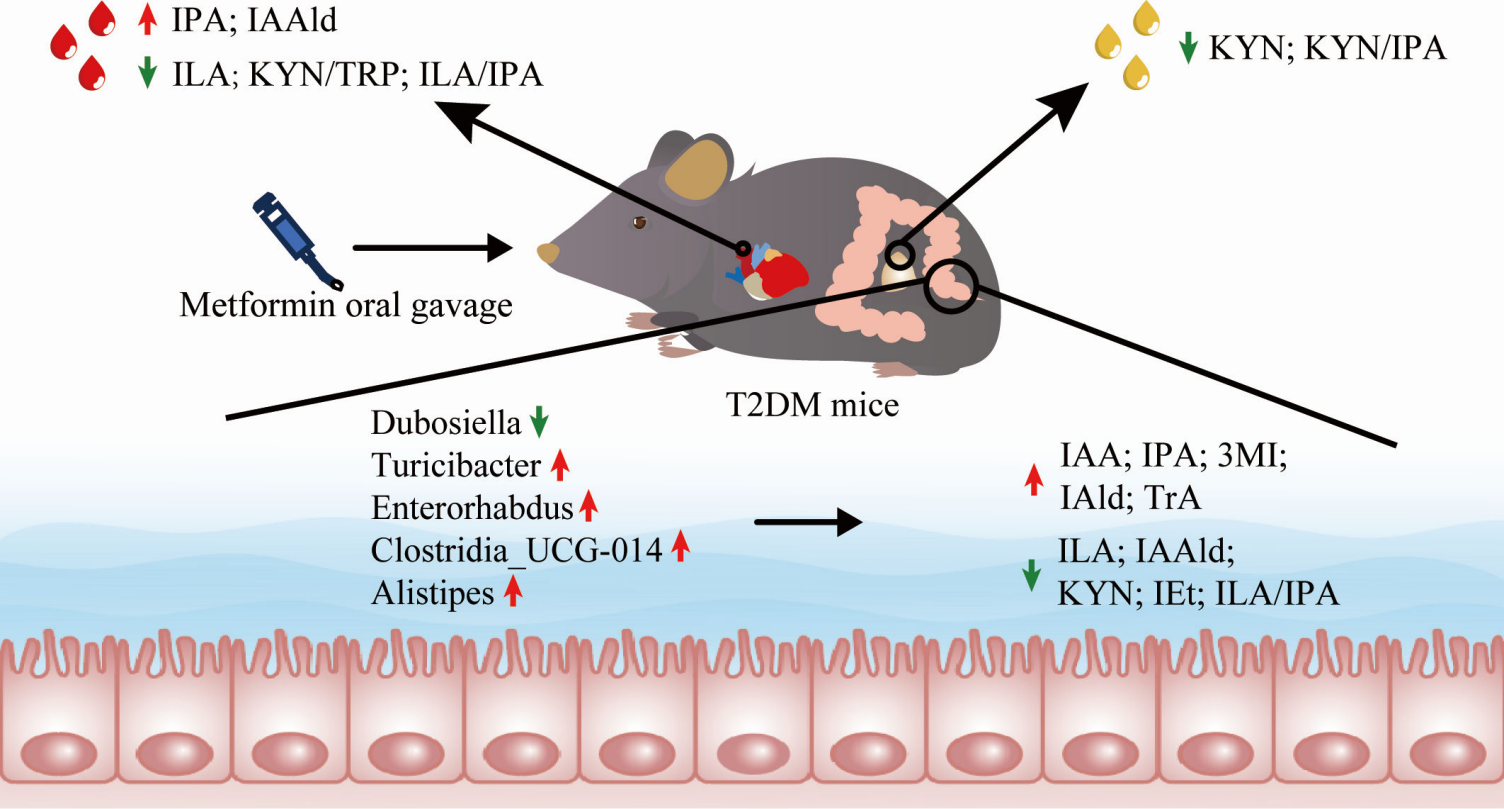
Schematic diagram illustrating the effect of metformin treatment on TRP metabolism spectrum in T2DM mice by modulating intestinal flora. After metformin treatment, the abundance of *Dubosiella*, *Turicibacter*, *Enterorhabdus*, *Clostridia UCG-014*, and *Alistipes* in T2DM mice was restored partially. While the concentrations of IAA, IPA, 3MI, IAld, and TrA were increased, and the concentrations of ILA, IAAld, KYN, IEt, and ILA/IPA were decreased. Besides, the concentrations of IPA, IAAld, ILA, and KYN/TRP, ILA/IPA were restored in serum, and KYN and ILA/IPA were restored in urine.

We successfully developed an LC-MS/MS method to quantify TRP and its 15 catabolites in biological samples. This method was validated and applied to assess TRP metabolism profiles in various biological samples from T2DM mice. As shown in Fig. 7, our findings unveiled perturbations in TRP metabolism in T2DM mice, predominantly characterized by increased ILA levels and decreased IPA levels in peripheral blood and intestinal contents, thus significantly disrupting the ILA/IPA ratio. Noteworthy, the ILA/IPA ratio reverted to normalcy post-treatment with metformin, suggesting the potential of ILA/IPA as a diagnostic marker or therapeutic target for T2DM. The regulation of TRP metabolism by metformin might involve the restoration of the relative abundance of *Turicibacter*, *Enterorhabdus*, RF39, *Clostridia*_UCG-014, and *Alistipes*. Additionally, the detection of indole metabolites in the serum of GF mice suggested the existence of endogenous TRP metabolic pathways in the host. Thus, the possibility of metformin modulating endogenous TRP metabolism should not be disregarded. Further investigations are required to unravel the intricate relationship between the gut microbiota, the host, and TRP metabolism.

## MATERIALS AND METHODS

### Chemicals and reagents

TRP, KYN, 3MI, TrA, ILA, Ind, IAld, IA, IPA, IAA, and 2-OX were obtained from Aladdin Bio-Technology Co., Ltd. (Shanghai, China). Indole-3-acetamide (IAM), IEt, and IPyA were purchased from Yuanye Bio-Technology Co., Ltd. (Shanghai, China). 5-HT and ISTD: Indole-3-acetic-2,2-d2 was purchased from Sigma-Aldrich (Shanghai, China). IAAld was purchased from Bide Pharmatech Ltd. (Shanghai, China). All reagents were obtained with a minimum purity of 97%, except IPyA (95%) and IAAld (90%). The chemical structures of the TRP metabolites are shown in Fig. S6. All organic solvents and water used in the sample and mobile phase preparations were of LC-MS grade and obtained from Sigma-Aldrich (Shanghai, China) and Watsons (Guangzhou, China).

### Preparation of stock solutions, ISTD mixture, and calibration curve standards

All 16 chemicals and the ISTD were weighed using a BT224S electronic balance (Sartorius AG, Göttingen, Germany). A stock solution of 10 mM in N, N-dimethylformamide was prepared for each standard, and the stock solution was diluted with 20% acetonitrile in the eight gradients (Table S1). All the solutions of each standard were stored at −20°C until use.

### Animal experiment design and sample collection

This study comprised three batches of mice experiments. (i) Five mice were housed in each cage and maintained at a temperature of 20 ± 2°C, in a 12-h diurnal cycle. They were provided with *ad libitum* access to food and water. At the conclusion of the experiment, fresh stool and urine samples were collected using sterile centrifuge tubes. Whole blood was kept at 24°C for 2 h, then centrifuged at 2,400 rpm at 4°C for 15 min to collect the supernatant. The mice were euthanized by cervical dislocation, and colonic contents were sampled. All samples were stored at −80°C. These SPF mouse samples were utilized for evaluating the precision and accuracy of the LC-MS/MS method.

1. Thirty-three-week-old male SPF C57BL/6J mice were purchased from SLAC Laboratory Animal Co., Ltd. This experiment was conducted at the Laboratory Animal Center of Zhejiang Academy of Agricultural Sciences and was approved by the Experimental Animal Ethics Committee of Zhejiang Province (approval number: 2021ZAASLA50). Upon arrival, the mice were acclimated to the animal chamber for 1 week in a 12-h diurnal cycle at a temperature of 24 ± 2°C, with unrestricted access to food and water. Subsequently, the mice were randomly divided into three groups: CON group, T2DM group, and MET group, with 10 mice in each group and 5 mice in each cage. The mice in the CON group were fed a normal maintenance diet, while the mice in the T2DM group and MET group were fed a customized high-fat and high-sugar diet (feed type: D12492, provided by Jiangsu Xetong Pharmaceutical Bioengineering Co., Ltd). After 6 weeks, the T2DM and MET mice were intraperitoneally injected with a streptozotocin solution at a dose of 80 mg/kg/day dissolved in a sodium citrate buffer (pH 4.2–4.5), whereas the CON group mice were intraperitoneally injected with sodium citrate buffer for 5 days. Once the model was successfully established, the mice in the MET group received metformin (100 mg/kg/day) via gavage for 6 weeks. Using the method established in this study, TRP metabolites were analyzed in the serum, colon contents, urine, and fecal samples of these groups of mice.
2. Blood and colon contents were sampled from three healthy, 3-week-old sterile Kunming mice that were utilized in our previous study (72). Additionally, 10 healthy, 6-week-old male conventional C57BL/6J mice were obtained from the Experimental Animal Center of Zhejiang Academy of Agricultural Sciences. All procedures were approved by the Third Military Medical University and Zhejiang Academy of Agricultural Sciences, respectively. This batch of mice was used to validate the indole pathway in germ-free mice.

### Sample extraction procedures

Serum samples were extracted as described by Xu et al. (73), with minor modifications. Then, 100 µL of the serum samples was mixed with 100 µL of the IS (1 µM) and 400 µL of methanol in a 1.5 mL centrifuge tube, vortexed for 10 s, and centrifuged at 4°C and 17,800 × *g* for 5 min. Subsequently, 450 µL of the supernatant was transferred into a new 1.5 mL centrifuge tube, and an additional 450 µL of methanol was added to the original centrifuge tube containing the precipitate. The mixture was vortexed, sonicated for 5 min, and then centrifuged at 4°C and 14,000 rpm for 5 min. The supernatant was collected and combined with the previous 450 µL supernatant solution. Finally, after centrifugation at 4°C and 14,000 rpm for 10 min, all the supernatant was aspirated, and the solution was filtered through a membrane.

The extraction method for the intestinal content and fecal samples was improved according to the method described by Zou et al. (20). Before sample processing, fresh samples were freeze-dried and ground into a powder. The sample (weighing 25 mg) was dissolved in a mixed solution of 250 µL water, 250 µL methanol, and 100 µL IS (1 µM), vortexed for 10 s, sonicated for 5 min, and centrifuged at 4°C and 17,800 × *g* for 5 min. Subsequently, 450 µL of the supernatant was transferred into a new 1.5 mL centrifuge tube, and an additional 450 µL of methanol was added to the original centrifuge tube containing the precipitate. The mixture was vortexed, sonicated for 5 min, and then centrifuged at 4°C and 14,000 rpm for 5 min. The supernatant was collected and combined with the previous 500 µL supernatant solution. Finally, after centrifugation at 4°C and 14,000 rpm for 10 min, all the supernatant was aspirated, and the solution was filtered through a membrane.

The extraction method for urine samples was slightly modified from that described by Zou et al. (20). In a 1.5 mL centrifuge tube, the sample (200 µL) was aspirated and mixed with 100 µL IS (1 µM) and 700 µL methanol. The solution was vortexed for 10 s, sonicated for 5 min, and placed at −20°C for 10 min. The solution was then centrifuged for 10 min at 4°C and 17,800 × *g*. Finally, 500 µL of supernatant was mixed with 500 µL of water, vortexed for 10 s, and observed to see whether it was clear. If not, centrifugation was repeated until the liquid was clear, after which the solution was filtered through a membrane.

### Instruments and LC-MS/MS conditions

LC-MS/MS analysis was performed using an AQUITY ultra-performance liquid chromatography system, thermostatic autosampler, ultra-high-performance binary pump (I-class, Waters, MA, USA), and a QTRAP 6500 tandem mass spectrometer (Sciex, Framingham, MA, USA). The controlling software was Analyst 1.6.2. Chromatographic separation was achieved on an ACQUITY PREMIER BEH C18 column (1.7 µm, 2.1 × 150 mm^2^, 1/pk, Waters, Milford, DE, USA) at 45°C. Considering the structural diversity and lipophilic range of the 16 compounds, mass spectrometric detection was performed using multiple reaction monitoring (MRM) with an electrospray ionization source in positive mode to minimize ion elution. For high-performance liquid chromatography, solvent A was 0.1% (vol/vol) formic acid in water, and solvent B was 0.1% (vol/vol) formic acid in ACN at a flow rate of 0.3 mL/min. The gradient elution procedure was as follows: 0–0.8 min, 85% A; 0.8–10.5 min, 85%–5% A; 10.5–11.4 min, 5% A; 11.4–11.5 min, 5%–85% A; and 11.5–12 min, 85% A.

The mass spectrometer was set to the positive electrospray ionization mode with MRM. The IonSpray voltage was set at 5,000 V and the temperature at 300°C. Curtain gas, ion source gas 1, ion source gas 2, and collision gas were set to 25, 10, 10, and medium, respectively. The entrance potential was set to 10 V. MRM transitions, declustering potential, collision energy, and collision cell exit potential were optimized using a syringe infusion pump. Table 1 summarizes the compound-specific chromatographic and mass spectrometry parameters, including retention time, MRM transitions, declustering potential, collision energy, and collision cell exit potential. Data acquisition and processing were performed using the Analyst software (version 1.6) from AB SCIEX.

### Method linearity, detection limit, and quantification limit

Sixteen analyte standard stock solutions were mixed with ISTD stock solutions, and the linearity of the calibration curves for each compound was evaluated under eight concentration gradients. Calibration curves were established using a linear regression equation and a linear correlation coefficient with the concentration and peak area. The LOD was defined as an *S*/*N* of 3:1, and the LOQ was defined as an *S*/*N* of 10:1.

### Method precision and accuracy QC

samples were prepared by mixing the ISTD and serum, feces from 10 specific pathogen-free mice to assess the recovery rate and precision. The intra-day and inter-day precision was evaluated by repeating the analysis of the QC samples on the same day and on three separate days. A daily calibration curve was used to calculate the concentration of each analyte in the sample, and the accuracy was expressed as the RSD.

### 16S rRNA sequencing and bioinformatic analysis

#### DNA extraction and PCR amplification

Microbial genomic DNA was extracted from the colon content according to the instructions (QIAamp DNA Stool Mini Kit, QIAGEN, CA, USA). The V4–V5 region of the bacteria 16S ribosomal RNA gene was amplified by PCR (95°C for 2 min, followed by 25 cycles at 95°C for 30 s, 55°C for 30 s, and 72°C for 30 s and a final extension at 72°C for 5 min) using primers 515 F (5′-GTGCCAGCMGCCGCGG-3′) and 907 R (5′-CCGTCAATTCMTTTRAGTTT-3′). Amplicons were extracted and purified using the AxyPrep DNA Gel Extraction Kit (Axygen Biosciences, Union City, CA, USA) and quantified using QuantiFluor-ST (Promega, USA).

#### Library construction and sequencing

The purified PCR products were quantified using Qubit 3.0 (Life Invitrogen), and 24 amplicons with different barcodes were mixed in equal proportions. The combined DNA products were utilized for constructing the Illumina Pair-End library following Illumina’s genomic DNA library preparation protocol. Subsequently, the amplicon library was subjected to paired-end sequencing (2 × 250) on a HiSeq 2500 PE250 platform [Mingke Biotechnology (Hangzhou) Co., Ltd] using standard procedures. The raw image data files obtained from high-throughput sequencing underwent Base Calling analysis to generate sequenced reads, which were then stored in FASTQ (fq) format files containing both sequence information and corresponding sequencing quality data.

#### Bioinformatic analysis

After sequencing, the raw fastq files underwent quality control and filtering, including the removal of tags and primers. The ASVs were clustered using the DADA2 package (version 2023.2.0) within QIIME2 (version 2023.2) software (74). For the filtering step, a truncation length of 370 (forward: 160, reverse: 210) was applied, along with a maximum expected error (maxEE) threshold of 2 and a truncation quality (truncq) of 2. Merging of the paired-end reads required a minimum overlap of 12. Taxonomic annotation of ASVs was conducted using the RDP Classifier against the SILVA database (version 138), with a confidence threshold of 0.7. And contaminant ASVs in the 16S rRNA gene sequencing data were identified using the decontam R package (version 1.22.0, based on DNA concentration) (75). Alpha-diversity and beta-diversity were performed by vegan (version 2.6.4) and ggsignif (version 0.6.4) package in R 4.3.1 software. The ropls (version 1.34.0) was utilized for the partial least squares discriminant analysis. NbClust (version 3.0.1) was utilized for the hierarchical cluster analysis. The LEfSe method (Performed using the OmicStudio tools at https://www.omicstudio.cn/tool/.) was used to identify each differentiated classification unit, and the threshold of the linear discriminant analysis score for discriminative features was set to 3.5. Functional prediction analysis was conducted using the q2-picrust2 plugin (version 2023.2). Spearman’s correlations (stats: version 4.3.1, pheatmap: version 1.0.12) were used to link the rich differential taxonomic groups with the TRP metabolites. Data were compared using the Wilcoxon rank-sum test, with a significance value of 0.05.

### Statistical analysis

SPSS software (version 26.0) was used for the statistical analysis. Statistical differences between the two groups were analyzed using an independent-sample *t* test, and data among the three groups were analyzed using one-way ANOVA with *post hoc* Bonferroni comparisons or Kruskal-Wallis with *post hoc* Bonferroni comparisons; *P* values <0.05 and <0.01 were considered statistically significant.

## Data Availability

The data sets generated in this study are openly available in the NCBI Sequence Read Archive (SRA) under BioProject accession number: PRJNA939340.

## SUPPLEMENTAL MATERIAL

Supplemental figures spectrum.00291-24-s0001.pdf Fig. S1 to S6.

Table S1 spectrum.00291-24-s0002.docx Preparation of standard solutions for standard curves.

ASM does not own the copyrights to Supplemental Material that may be linked to, or accessed through, an article. The authors have granted ASM a non-exclusive, world-wide license to publish the Supplemental Material files. Please contact the corresponding author directly for reuse.

## References

[1] Cunningham AL, Stephens JW, Harris DA. 2021. Gut microbiota influence in type 2 diabetes mellitus (T2DM). Gut Pathog 13:50. doi:10.1186/s13099-021-00446-0 34362432 PMC8343927

[2] Di Mauro S, Filippello A, Scamporrino A, Purrello F, Piro S, Malaguarnera R. 2022. Metformin: when should we fear lactic acidosis? Int J Mol Sci 23:8320. doi:10.3390/ijms23158320 35955455 PMC9368510

[3] Dutta S, Shah RB, Singhal S, Dutta SB, Bansal S, Sinha S, Haque M. 2023. Metformin: a review of potential mechanism and therapeutic utility beyond diabetes. Drug Des Devel Ther 17:1907–1932. doi:10.2147/DDDT.S409373 PMC10312383 37397787

[4] Wu H, Esteve E, Tremaroli V, Khan MT, Caesar R, Mannerås-Holm L, Ståhlman M, Olsson LM, Serino M, Planas-Fèlix M, Xifra G, Mercader JM, Torrents D, Burcelin R, Ricart W, Perkins R, Fernàndez-Real JM, Bäckhed F. 2017. Metformin alters the gut microbiome of individuals with treatment-naive type 2 diabetes, contributing to the therapeutic effects of the drug. Nat Med 23:850–858. doi:10.1038/nm.4345 28530702

[5] Karlsson FH, Tremaroli V, Nookaew I, Bergström G, Behre CJ, Fagerberg B, Nielsen J, Bäckhed F. 2013. Gut metagenome in European women with normal, impaired and diabetic glucose control. Nature 498:99–103. doi:10.1038/nature12198 23719380

[6] Agus A, Planchais J, Sokol H. 2018. Gut microbiota regulation of tryptophan metabolism in health and disease. Cell Host Microbe 23:716–724. doi:10.1016/j.chom.2018.05.003 29902437

[7] Masse KE, Lu VB. 2023. Short-chain fatty acids, secondary bile acids and indoles: gut microbial metabolites with effects on enteroendocrine cell function and their potential as therapies for metabolic disease. Front Endocrinol (Lausanne) 14:1169624. doi:10.3389/fendo.2023.1169624 37560311 PMC10407565

[8] Ma L, Tao S, Song T, Lyu W, Li Y, Wang W, Shen Q, Ni Y, Zhu J, Zhao J, Yang H, Xiao Y. 2024. Clostridium butyricum and carbohydrate active enzymes contribute to the reduced fat deposition in pigs. iMeta 3:e160. doi:10.1002/imt2.160 38868506 PMC10989082

[9] Kwan SY, Sabotta CM, Joon A, Wei P, Petty LE, Below JE, Wu X, Zhang J, Jenq RR, Hawk ET, McCormick JB, Fisher-Hoch SP, Beretta L. 2022. Gut microbiome alterations associated with diabetes in mexican americans in South Texas. mSystems 7:e0003322. doi:10.1128/msystems.00033-22 35477306 PMC9238400

[10] Gaike AH, Paul D, Bhute S, Dhotre DP, Pande P, Upadhyaya S, Reddy Y, Sampath R, Ghosh D, Chandraprabha D, Acharya J, Banerjee G, Sinkar VP, Ghaskadbi SS, Shouche YS. 2020. The gut microbial diversity of newly diagnosed diabetics but not of prediabetics is significantly different from that of healthy nondiabetics. mSystems 5:e00578-19. doi:10.1128/mSystems.00578-19 32234773 PMC7112960

[11] Foretz M, Guigas B, Viollet B. 2023. Metformin: update on mechanisms of action and repurposing potential. Nat Rev Endocrinol 19:460–476. doi:10.1038/s41574-023-00833-4 37130947 PMC10153049

[12] Sun L, Xie C, Wang G, Wu Y, Wu Q, Wang X, Liu J, Deng Y, Xia J, Chen B, et al.. 2018. Gut microbiota and intestinal FXR mediate the clinical benefits of metformin. Nat Med 24:1919–1929. doi:10.1038/s41591-018-0222-4 30397356 PMC6479226

[13] Qi Q, Li J, Yu B, Moon J-Y, Chai JC, Merino J, Hu J, Ruiz-Canela M, Rebholz C, Wang Z, et al.. 2022. Host and gut microbial tryptophan metabolism and type 2 diabetes: an integrative analysis of host genetics, diet, gut microbiome and circulating metabolites in cohort studies. Gut 71:1095–1105. doi:10.1136/gutjnl-2021-324053 34127525 PMC8697256

[14] Gürcü S, Girgin G, Yorulmaz G, Kılıçarslan B, Efe B, Baydar T. 2020. Neopterin and biopterin levels and tryptophan degradation in patients with diabetes. Sci Rep 10:17025. doi:10.1038/s41598-020-74183-w 33046801 PMC7552423

[15] de Mello VD, Paananen J, Lindström J, Lankinen MA, Shi L, Kuusisto J, Pihlajamäki J, Auriola S, Lehtonen M, Rolandsson O, Bergdahl IA, Nordin E, Ilanne-Parikka P, Keinänen-Kiukaanniemi S, Landberg R, Eriksson JG, Tuomilehto J, Hanhineva K, Uusitupa M. 2017. Indolepropionic acid and novel lipid metabolites are associated with a lower risk of type 2 diabetes in the finnish diabetes prevention study. Sci Rep 7:46337. doi:10.1038/srep46337 28397877 PMC5387722

[16] Tuomainen M, Lindström J, Lehtonen M, Auriola S, Pihlajamäki J, Peltonen M, Tuomilehto J, Uusitupa M, de Mello VD, Hanhineva K. 2018. Associations of serum indolepropionic acid, a gut microbiota metabolite, with type 2 diabetes and low-grade inflammation in high-risk individuals. Nutr Diabetes 8:35. doi:10.1038/s41387-018-0046-9 29795366 PMC5968030

[17] Shi Z, Lei H, Chen G, Yuan P, Cao Z, Ser H-L, Zhu X, Wu F, Liu C, Dong M, Song Y, Guo Y, Chen C, Hu K, Zhu Y, Zeng X-A, Zhou J, Lu Y, Patterson AD, Zhang L. 2021. Impaired intestinal Akkermansia muciniphila and aryl hydrocarbon receptor ligands contribute to nonalcoholic fatty liver disease in mice. mSystems 6:e00985-20. doi:10.1128/mSystems.00985-20 33622853 PMC8573958

[18] Jacob M, Malkawi A, Albast N, Al Bougha S, Lopata A, Dasouki M, Abdel Rahman AM. 2018. A targeted metabolomics approach for clinical diagnosis of inborn errors of metabolism. Anal Chim Acta 1025:141–153. doi:10.1016/j.aca.2018.03.058 29801603

[19] Lai Y, Liu CW, Chi L, Ru H, Lu K. 2021. high-resolution metabolomics of 50 neurotransmitters and tryptophan metabolites in feces, serum, and brain tissues using UHPLC-ESI-Q exactive mass spectrometry. ACS Omega 6:8094–8103. doi:10.1021/acsomega.0c05789 33817468 PMC8014936

[20] Zou B, Sun Y, Xu Z, Chen Y, Li L, Lin L, Zhang S, Liao Q, Xie Z. 2021. Rapid simultaneous determination of gut microbial phenylalanine, tyrosine, and tryptophan metabolites in rat serum, urine, and faeces using LC-MS/MS and its application to a type 2 diabetes mellitus study. Biomed Chromatogr 35:e4985. doi:10.1002/bmc.4985 33200425

[21] Dong F, Hao F, Murray IA, Smith PB, Koo I, Tindall AM, Kris-Etherton PM, Gowda K, Amin SG, Patterson AD, Perdew GH. 2020. Intestinal microbiota-derived tryptophan metabolites are predictive of Ah receptor activity. Gut Microbes 12:1–24. doi:10.1080/19490976.2020.1788899 PMC7524359 32783770

[22] Marcobal A, Kashyap PC, Nelson TA, Aronov PA, Donia MS, Spormann A, Fischbach MA, Sonnenburg JL. 2013. A metabolomic view of how the human gut microbiota impacts the host metabolome using humanized and gnotobiotic mice. ISME J 7:1933–1943. doi:10.1038/ismej.2013.89 23739052 PMC3965317

[23] Wang LS, Zhang MD, Tao X, Zhou YF, Liu XM, Pan RL, Liao YH, Chang Q. 2019. LC-MS/MS-based quantification of tryptophan metabolites and neurotransmitters in the serum and brain of mice. J Chromatogr B Analyt Technol Biomed Life Sci 1112:24–32. doi:10.1016/j.jchromb.2019.02.021 30836315

[24] Panitz V, Končarević S, Sadik A, Friedel D, Bausbacher T, Trump S, Farztdinov V, Schulz S, Sievers P, Schmidt S, et al.. 2021. Tryptophan metabolism is inversely regulated in the tumor and blood of patients with glioblastoma. Theranostics 11:9217–9233. doi:10.7150/thno.60679 34646367 PMC8490504

[25] Roager HM, Licht TR. 2018. Microbial tryptophan catabolites in health and disease. Nat Commun 9:3294–3303. doi:10.1038/s41467-018-05470-4 30120222 PMC6098093

[26] Li X, Zhang B, Hu Y, Zhao Y. 2021. New insights into gut-bacteria-derived indole and its derivatives in intestinal and liver diseases. Front Pharmacol 12:769501. doi:10.3389/fphar.2021.769501 34966278 PMC8710772

[27] Wyatt M, Greathouse KL. 2021. Targeting dietary and microbial tryptophan-indole metabolism as therapeutic approaches to colon cancer. Nutrients 13:1189–1211. doi:10.3390/nu13041189 33916690 PMC8066279

[28] Aoki R, Aoki-Yoshida A, Suzuki C, Takayama Y. 2018. Indole-3-pyruvic acid, an aryl hydrocarbon receptor activator, suppresses experimental colitis in mice. J Immunol 201:3683–3693. doi:10.4049/jimmunol.1701734 30429284

[29] Liu J-R, Miao H, Deng D-Q, Vaziri ND, Li P, Zhao Y-Y. 2021. Gut microbiota-derived tryptophan metabolism mediates renal fibrosis by aryl hydrocarbon receptor signaling activation. Cell. Mol. Life Sci 78:909–922. doi:10.1007/s00018-020-03645-1 32965514 PMC11073292

[30] Forslund K, Hildebrand F, Nielsen T, Falony G, Le Chatelier E, Sunagawa S, Prifti E, Vieira-Silva S, Gudmundsdottir V, Krogh Pedersen H, et al.. 2015. Disentangling type 2 diabetes and metformin treatment signatures in the human gut microbiota. Nature 528:262–266. doi:10.1038/nature15766 26633628 PMC4681099

[31] Sadok I, Rachwał K, Staniszewska M. 2019. Application of the optimized and validated LC-MS method for simultaneous quantification of tryptophan metabolites in culture medium from cancer cells. J Pharm Biomed Anal 176:112805. doi:10.1016/j.jpba.2019.112805 31415991

[32] Takahashi S, Iizuka H, Kuwabara R, Naito Y, Sakamoto T, Miyagi A, Onozato M, Ichiba H, Fukushima T. 2016. Determination of l-tryptophan and l-kynurenine derivatized with (R)-4-(3-isothiocyanatopyrrolidin-1-yl)-7-(N,N-dimethylaminosulfonyl)-2,1,3-benzoxadiazole by LC-MS/MS on a triazole-bonded column and their quantification in human serum. Biomed Chromatogr 30:1481–1486. doi:10.1002/bmc.3709 26910189

[33] Hu L-J, Li X-F, Hu J-Q, Ni X-J, Lu H-Y, Wang J-J, Huang X-N, Lin C-X, Shang D-W, Wen Y-G. 2017. A simple HPLC-ms/ms method for determination of tryptophan, kynurenine and kynurenic acid in human serum and its potential for monitoring antidepressant therapy. J Anal Toxicol 41:37–44. doi:10.1093/jat/bkw071 27590037

[34] van Zundert SKM, Griffioen PH, van Rossem L, Willemsen SP, de Rijke YB, van Schaik RHN, Steegers-Theunissen RPM, Mirzaian M. 2023. Simultaneous quantification of tryptophan metabolites by liquid chromatography tandem mass spectrometry during early human pregnancy. Clin Chem Lab Med 61:442–451. doi:10.1515/cclm-2022-0790 36458576

[35] Pedraz-Petrozzi B, Marszalek-Grabska M, Kozub A, Szalaj K, Trzpil A, Stachniuk A, Lamadé EK, Gilles M, Deuschle M, Turski WA, Fornal E. 2023. LC–MS/MS-based quantification of tryptophan, kynurenine, and kynurenic acid in human placental, fetal membranes, and umbilical cord samples. Sci Rep 13:12554–12563. doi:10.1038/s41598-023-39774-3 37532780 PMC10397233

[36] US Department of Health and Human Services FaDA, Center for Drug Evaluation and Research (CDER), Center for Veterinary Medicine (CV). 2018. Bioanalytical method validation guidance for industry.

[37] Lin HV, Efanov AM, Fang X, Beavers LS, Wang X, Wang J, Gonzalez Valcarcel IC, Ma T. 2016. GPR142 controls tryptophan-induced insulin and incretin hormone secretion to improve glucose metabolism. PLoS One 11:e0157298. doi:10.1371/journal.pone.0157298 27322810 PMC4920590

[38] Ueda Y, Iwakura H, Bando M, Doi A, Ariyasu H, Inaba H, Morita S, Akamizu T. 2018. Differential role of GPR142 in tryptophan-mediated enhancement of insulin secretion in obese and lean mice. PLoS One 13:e0198762. doi:10.1371/journal.pone.0198762 29889885 PMC5995358

[39] Matsuoka K, Kato K, Takao T, Ogawa M, Ishii Y, Shimizu F, Masuda J, Takada A. 2017. Concentrations of various tryptophan metabolites are higher in patients with diabetes mellitus than in healthy aged male adults. Diabetol Int 8:69–75. doi:10.1007/s13340-016-0282-y 30603309 PMC6224928

[40] Lanser L, Kink P, Egger EM, Willenbacher W, Fuchs D, Weiss G, Kurz K. 2020. Inflammation-induced tryptophan breakdown is related with anemia, fatigue, and depression in cancer. Front Immunol 11:249. doi:10.3389/fimmu.2020.00249 32153576 PMC7047328

[41] Benech N, Rolhion N, Sokol H. 2022. Gut microbiota reprogramming of tryptophan metabolism during pregnancy shapes host insulin resistance. Gastroenterology 162:1587–1589. doi:10.1053/j.gastro.2022.01.059 35247461

[42] Pedersen ER, Svingen GFT, Schartum-Hansen H, Ueland PM, Ebbing M, Nordrehaug JE, Igland J, Seifert R, Nilsen RM, Nygård O. 2013. Urinary excretion of kynurenine and tryptophan, cardiovascular events, and mortality after elective coronary angiography. Eur Heart J 34:2689–2696. doi:10.1093/eurheartj/eht264 23886918

[43] Oxenkrug G. 2013. Insulin resistance and dysregulation of tryptophan-kynurenine and kynurenine-nicotinamide adenine dinucleotide metabolic pathways. Mol Neurobiol 48:294–301. doi:10.1007/s12035-013-8497-4 23813101 PMC3779535

[44] Abedi S, Vessal M, Asadian F, Takhshid MA. 2021. Association of serum kynurenine/tryptophan ratio with poor glycemic control in patients with type2 diabetes. J Diabetes Metab Disord 20:1521–1527. doi:10.1007/s40200-021-00895-z 34900804 PMC8630152

[45] Du L, Qi R, Wang J, Liu Z, Wu Z. 2021. Indole-3-propionic acid, a functional metabolite of Clostridium sporogenes, promotes muscle tissue development and reduces muscle cell inflammation. Int J Mol Sci 22:12435–12450. doi:10.3390/ijms222212435 34830317 PMC8619491

[46] Serger E, Luengo-Gutierrez L, Chadwick JS, Kong G, Zhou L, Crawford G, Danzi MC, Myridakis A, Brandis A, Bello AT, Müller F, Sanchez-Vassopoulos A, De Virgiliis F, Liddell P, Dumas ME, Strid J, Mani S, Dodd D, Di Giovanni S. 2022. The gut metabolite indole-3 propionate promotes nerve regeneration and repair. Nature 607:585–592. doi:10.1038/s41586-022-04884-x 35732737

[47] Sedighi M, Razavi S, Navab-Moghadam F, Khamseh ME, Alaei-Shahmiri F, Mehrtash A, Amirmozafari N. 2017. Comparison of gut microbiota in adult patients with type 2 diabetes and healthy individuals. Microb Pathog 111:362–369. doi:10.1016/j.micpath.2017.08.038 28912092

[48] Zelante T, Iannitti RG, Cunha C, De Luca A, Giovannini G, Pieraccini G, Zecchi R, D’Angelo C, Massi-Benedetti C, Fallarino F, Carvalho A, Puccetti P, Romani L. 2013. Tryptophan catabolites from microbiota engage aryl hydrocarbon receptor and balance mucosal reactivity via interleukin-22. Immunity 39:372–385. doi:10.1016/j.immuni.2013.08.003 23973224

[49] Sameera B, Prakash H. 2018. Indole acetic acid production by the actinomycetes of coffee plantation soils. Int J Curr Res 10:74482–74487.

[50] Kaakoush NO. 2015. Insights into the role of erysipelotrichaceae in the human host. Front Cell Infect Microbiol 5:84. doi:10.3389/fcimb.2015.00084 26636046 PMC4653637

[51] Salomäki-Myftari H, Vähätalo LH, Ailanen L, Pietilä S, Laiho A, Hänninen A, Pursiheimo J-P, Munukka E, Rintala A, Savontaus E, Pesonen U, Koulu M. 2016. Neuropeptide Y overexpressing female and male mice show divergent metabolic but not gut microbial responses to prenatal metformin exposure. PLoS One 11:e0163805. doi:10.1371/journal.pone.0163805 27681875 PMC5040270

[52] Guo X, Cao X, Fang X, Guo A, Li E. 2021. Inhibitory effects of fermented Ougan (Citrus reticulata cv. Suavissima) juice on high-fat diet-induced obesity associated with white adipose tissue browning and gut microbiota modulation in mice. Food Funct 12:9300–9314. doi:10.1039/d0fo03423a 34606525

[53] Wang Y, Li L, Ye C, Yuan J, Qin S. 2020. Alginate oligosaccharide improves lipid metabolism and inflammation by modulating gut microbiota in high-fat diet fed mice. Appl Microbiol Biotechnol 104:3541–3554. doi:10.1007/s00253-020-10449-7 32103315

[54] Broadfield LA, Saigal A, Szamosi JC, Hammill JA, Bezverbnaya K, Wang D, Gautam J, Tsakiridis EE, Di Pastena F, McNicol J, et al.. 2022. Metformin-induced reductions in tumor growth involves modulation of the gut microbiome. Mol Metab 61:101498. doi:10.1016/j.molmet.2022.101498 35452877 PMC9096669

[55] Fernandez-Quintela A, Macarulla MT, Gómez-Zorita S, González M, Milton-Laskibar I, Portillo MP. 2022. Relationship between changes in microbiota induced by resveratrol and its anti-diabetic effect on type 2 diabetes. Front Nutr 9:1084702. doi:10.3389/fnut.2022.1084702 36687699 PMC9852824

[56] Zhang F, Wang M, Yang J, Xu Q, Liang C, Chen B, Zhang J, Yang Y, Wang H, Shang Y, Wang Y, Mu X, Zhu D, Zhang C, Yao M, Zhang L. 2019. Response of gut microbiota in type 2 diabetes to hypoglycemic agents. Endocrine 66:485–493. doi:10.1007/s12020-019-02041-5 31410749

[57] Lynch JB, Gonzalez EL, Choy K, Faull KF, Jewell T, Arellano A, Liang J, Yu KB, Paramo J, Hsiao EY. 2023. Gut microbiota Turicibacter strains differentially modify bile acids and host lipids. Nat Commun 14:3669. doi:10.1038/s41467-023-39403-7 37339963 PMC10281990

[58] Pirozzi C, Coretti L, Opallo N, Bove M, Annunziata C, Comella F, Turco L, Lama A, Trabace L, Meli R, Lembo F, Mattace Raso G. 2023. Palmitoylethanolamide counteracts high-fat diet-induced gut dysfunction by reprogramming microbiota composition and affecting tryptophan metabolism. Front Nutr 10:1143004. doi:10.3389/fnut.2023.1143004 37599675 PMC10434518

[59] Fung TC, Vuong HE, Luna CDG, Pronovost GN, Aleksandrova AA, Riley NG, Vavilina A, McGinn J, Rendon T, Forrest LR, Hsiao EY. 2019. Intestinal serotonin and fluoxetine exposure modulate bacterial colonization in the gut. Nat Microbiol 4:2064–2073. doi:10.1038/s41564-019-0540-4 31477894 PMC6879823

[60] Jiang H, Ling Z, Zhang Y, Mao H, Ma Z, Yin Y, Wang W, Tang W, Tan Z, Shi J, Li L, Ruan B. 2015. Altered fecal microbiota composition in patients with major depressive disorder. Brain Behav Immun 48:186–194. doi:10.1016/j.bbi.2015.03.016 25882912

[61] Zhang C, Yin A, Li H, Wang R, Wu G, Shen J, Zhang M, Wang L, Hou Y, Ouyang H, et al.. 2015. Dietary modulation of gut microbiota contributes to alleviation of both genetic and simple obesity in children. EBioMed 2:968–984. doi:10.1016/j.ebiom.2015.07.007 PMC4563136 26425705

[62] Menni C, Hernandez MM, Vital M, Mohney RP, Spector TD, Valdes AM. 2019. Circulating levels of the anti-oxidant indoleproprionic acid are associated with higher gut microbiome diversity. Gut Microbes 10:688–695. doi:10.1080/19490976.2019.1586038 31030641 PMC6866703

[63] Lagkouvardos I, Pukall R, Abt B, Foesel BU, Meier-Kolthoff JP, Kumar N, Bresciani A, Martínez I, Just S, Ziegler C, et al.. 2016. The mouse intestinal bacterial collection (miBC) provides host-specific insight into cultured diversity and functional potential of the gut microbiota. Nat Microbiol 1. doi:10.1038/nmicrobiol.2016.131 27670113

[64] Zarei I, Koistinen VM, Kokla M, Klåvus A, Babu AF, Lehtonen M, Auriola S, Hanhineva K. 2022. Tissue-wide metabolomics reveals wide impact of gut microbiota on mice metabolite composition. Sci Rep 12:15018. doi:10.1038/s41598-022-19327-w 36056162 PMC9440220

[65] Shimada Y, Kinoshita M, Harada K, Mizutani M, Masahata K, Kayama H, Takeda K. 2013. Commensal bacteria-dependent indole production enhances epithelial barrier function in the colon. PLoS One 8:e80604. doi:10.1371/journal.pone.0080604 24278294 PMC3835565

[66] Gao J, Xu K, Liu H, Liu G, Bai M, Peng C, Li T, Yin Y. 2018. Impact of the gut microbiota on intestinal immunity mediated by tryptophan metabolism. Front Cell Infect Microbiol 8:13. doi:10.3389/fcimb.2018.00013 29468141 PMC5808205

[67] Whitt DD, Demoss RD. 1975. Effect of microflora on the free amino acid distribution in various regions of the mouse gastrointestinal tract. Appl Microbiol 30:609–615. doi:10.1128/am.30.4.609-615.1975 1190761 PMC187239

[68] Roager HM, Licht TR. 2018. Microbial tryptophan catabolites in health and disease. Nat Commun 9. doi:10.1038/s41467-018-05470-4 PMC6098093 30120222

[69] Run L, Tian Z, Xu L, Du J, Li N, Wang Q, Sun H. 2023. Knockdown of IL4I1 improved high glucose-evoked insulin resistance in HepG2 cells by alleviating inflammation and lipotoxicity through AHR activation. Appl Biochem Biotechnol 195:6694–6707. doi:10.1007/s12010-023-04399-9 36913096

[70] Sadik A, Somarribas Patterson LF, Öztürk S, Mohapatra SR, Panitz V, Secker PF, Pfänder P, Loth S, Salem H, Prentzell MT, et al.. 2020. IL4I1 Is a metabolic immune checkpoint that activates the AHR and promotes tumor progression. Cell 182:1252–1270. doi:10.1016/j.cell.2020.07.038 32818467

[71] Zhang X, Gan M, Li J, Li H, Su M, Tan D, Wang S, Jia M, Zhang L, Chen G. 2020. Endogenous indole pyruvate pathway for tryptophan metabolism mediated b IL4I1. J Agric Food Chem 68:10678–10684. doi:10.1021/acs.jafc.0c03735 32866000

[72] Yin Y, Li M, Gu W, Zeng B, Liu W, Zhu L, Pi X, Primerano DA, Yu HD, Wei H, Yu G, Wang X. 2021. Carrageenan oligosaccharides and associated carrageenan-degrading bacteria induce intestinal inflammation in germ-free mice. J Genet Genomics 48:815–824. doi:10.1016/j.jgg.2021.08.001 34400364 PMC8628850

[73] Xu H, Pan L-B, Yu H, Han P, Fu J, Zhang Z-W, Hu J-C, Yang X-Y, Keranmu A, Zhang H-J, Bu M-M, Jiang J-D, Wang Y. 2022. Gut microbiota-derived metabolites in inflammatory diseases based on targeted metabolomics. Front Pharmacol 13:919181. doi:10.3389/fphar.2022.919181 36238574 PMC9551995

[74] Wang S, Tian ZB, Chen JW, Cong PS, Ding XL, Zhang CP, Yin XY, Yang L, Jing X, Mao T, Li XY, Sun ZY, Jiang JJ, Yu YN. 2023. Effect of fucoidan on gut microbiota and its clinical efficacy in Helicobacter pylori eradication: a randomized controlled trial. J Digest Diseases 24:461–471. doi:10.1111/1751-2980.13215 37548312

[75] Davis NM, Proctor DM, Holmes SP, Relman DA, Callahan BJ. 2018. Simple statistical identification and removal of contaminant sequences in marker-gene and metagenomics data. Microbiome 6:226. doi:10.1186/s40168-018-0605-2 30558668 PMC6298009

