# Supplementary material for: Interplay between gut microbiota and tryptophan metabolism in type 2 diabetic mice treated with metformin
Source: Microbiol Spectr. 2024 Aug 20;12(10):e00291-24. doi: 10.1128/spectrum.00291-24 (PMC11448047; doi:10.1128/spectrum.00291-24)
Supplement: Supplemental figures — Fig. S1 to S6. [file spectrum.00291-24-s0001.pdf]

Supplementary Figures

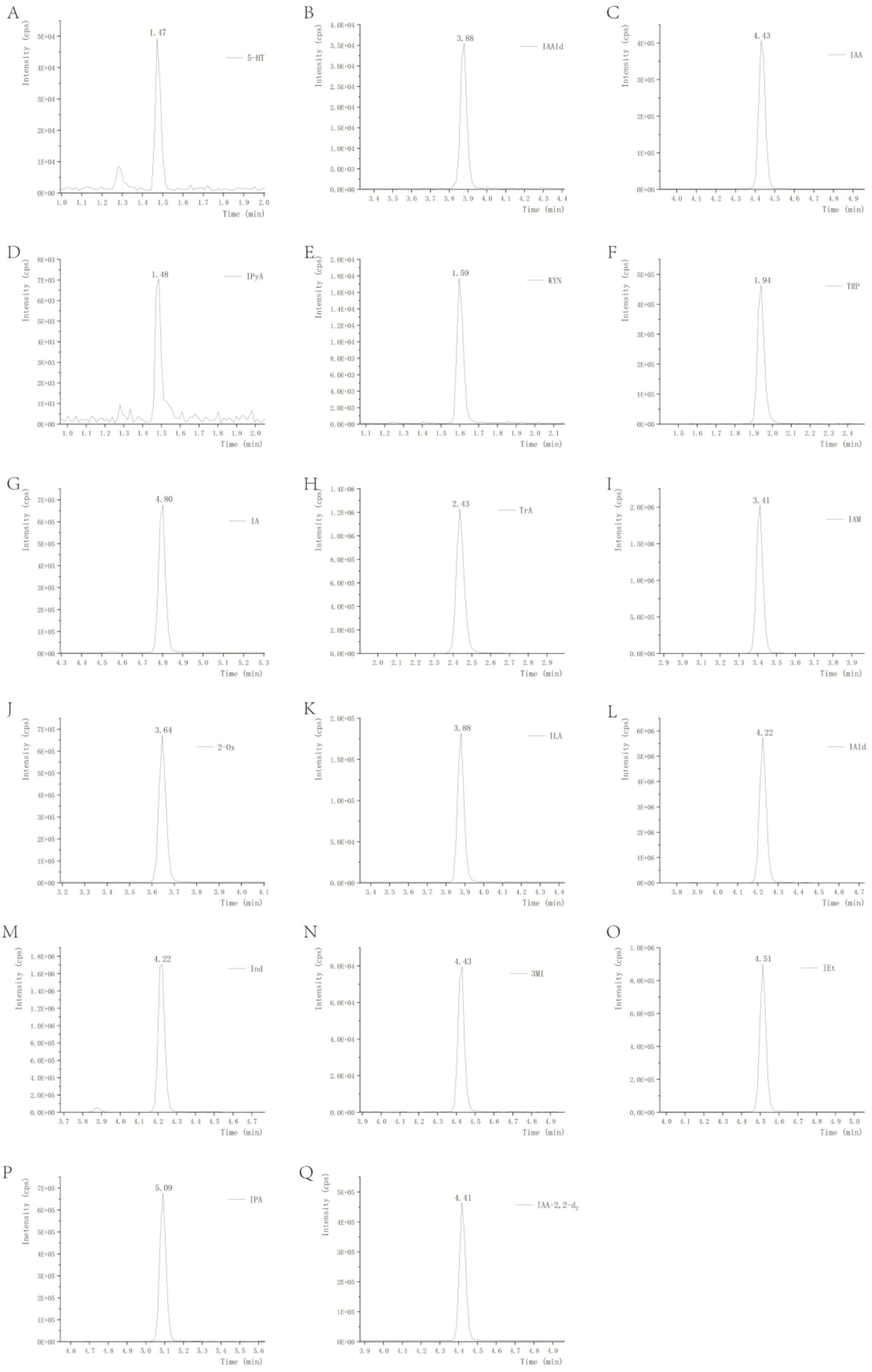

Fig. S1: Individual MRM chromatogram of 16 metabolites and IS optimized by LC-MS/MS. List of compounds: A: 5-hydroxytryptamine (5-HT); B: Indole-3-acetaldehyde (IAAld); C: Indole-3-acetic acid (IAA); D: Indole-3-pyruvic acid (IPyA); E: Kynurenine (KYN); F: L-Tryptophan (TRP); G: Indole-3-acrylic acid (IA); H: Tryptamine (TrA); I: Indole-3-acetamide (IAM); J: 2-Oxindole (2-Ox); K: Indole-3-lactic acid (ILA); L: Indole-3-aldehyde (IAld); M: Indole (Ind); N: 3-Skatole (3MI); O: Tryptophol (IEt); P: Indole-3-propionic acid (IPA); Q: Indole-3-acetic-2,2-d2 (IAA-2,2-d2).

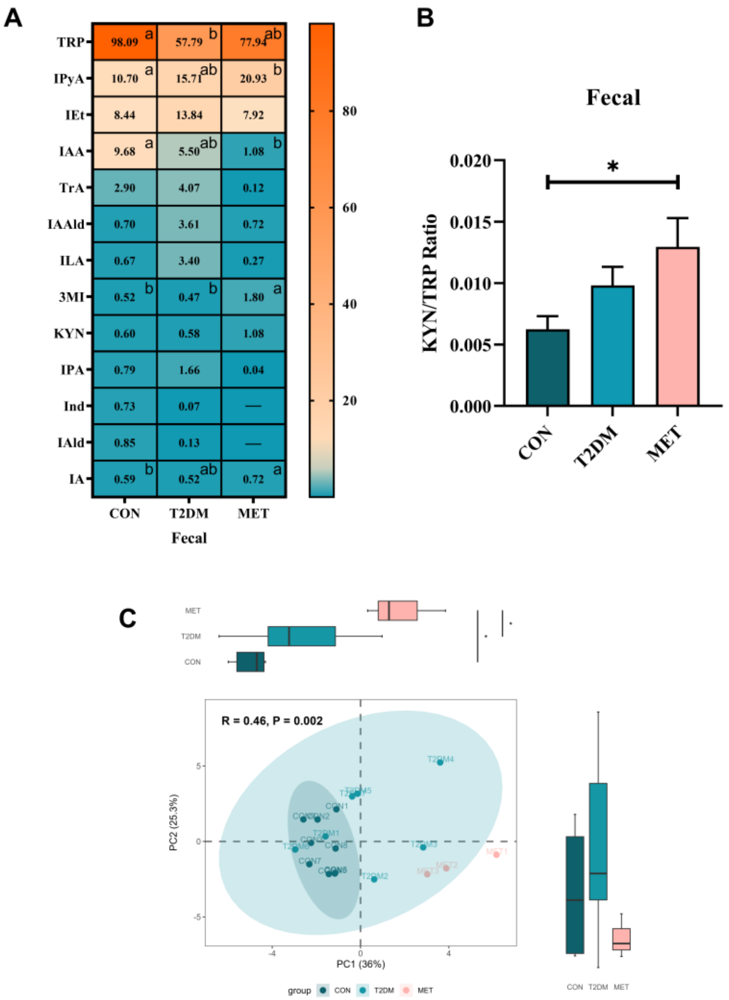

Fig. S2: T2DM and metformin treatment modified mice fecal tryptophan metabolism. The concentration of TRP and its metabolites in fecal samples (A, n=3-9) were determined by LC-MS/MS. The ratio of KYN to TRP in fecal samples (B). Principal component analysis of TRP and its metabolites in fecal samples (C). The heatmap illustrates the mean values, with redder colors indicating higher values and bluer colors indicating lower values. The data in histograms are expressed as the mean ± SEM. Statistical differences in the heatmaps and histograms were analyzed using one-way ANONA with post hoc comparisons using Bonferroni. In the heatmap, different letters indicate significant differences ($p < 0.05$), and significant differences in the histograms are denoted by (*) for $p < 0.05$ and (**) for $p < 0.01$. Principal component analysis of the metabolites in fecal samples (C) based on bray-curtis distance, anosim was used to calculate R and *p* values.

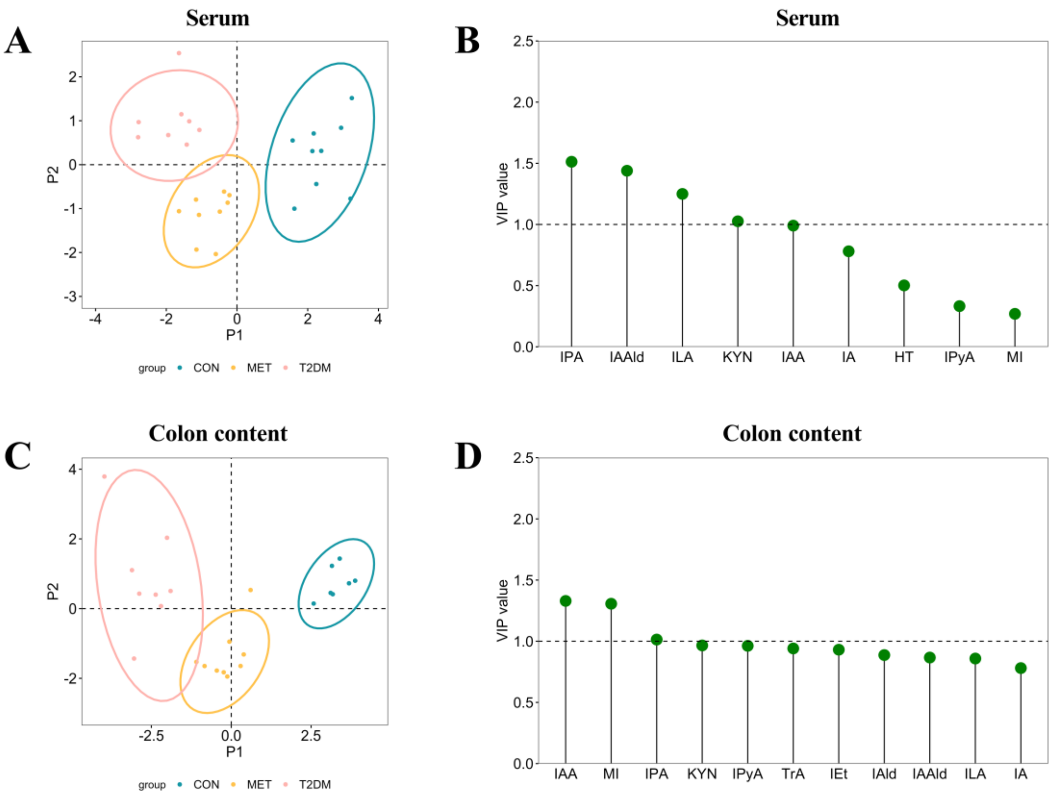

Fig. S3: The analysis of TRP metabolites in serum (A) and colonic contents (C) using partial least squares discriminant analysis, as well as the variable importance projection scores for TRP metabolites in serum (B) and colonic contents (D).

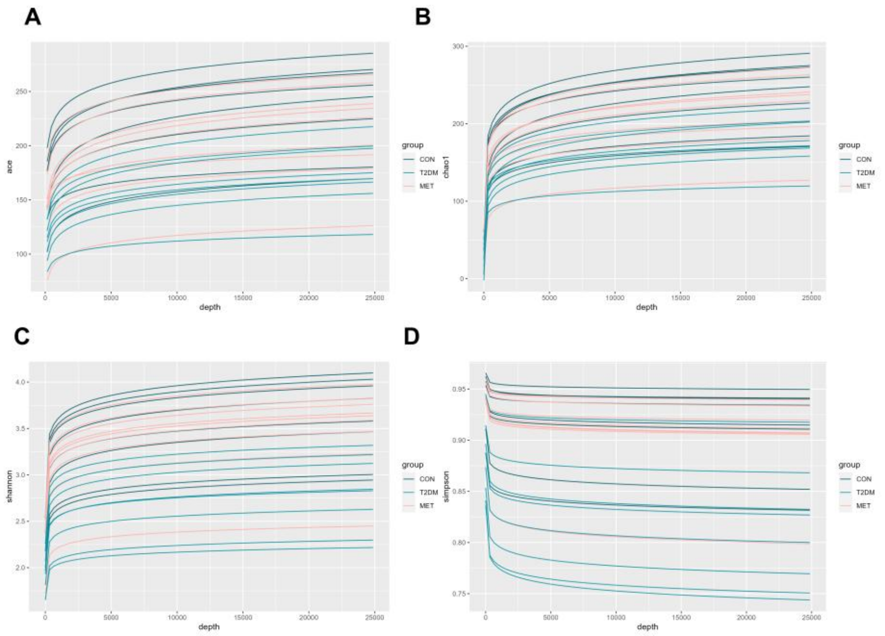

Fig. S4: Rarefaction curves of colonic contents flora from CON, T2DM and MET mice. The curve of ACE (A), Chao1 index (B), Shannon index (C) and Simpson index of different samples or groups changing with leveling depth.

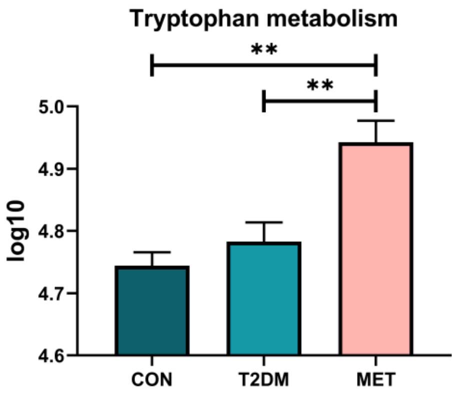

Fig. S5: Metformin therapy enriched genes related to tryptophan metabolism in the intestinal flora. Colon contents of three groups of mice (n=8-9) were collected for 16S rRNA Sequencing. PICRUST2 was used to predict the function of sequencing results. Tryptophan metabolism related gene content at the tertiary classification level of KEGG. Statistical differences in histograms were analyzed by one-way ANOVA with post hoc comparisons using Bonferroni. Significant difference in histograms with $p < 0.05$ (*), $p < 0.01$ (**).

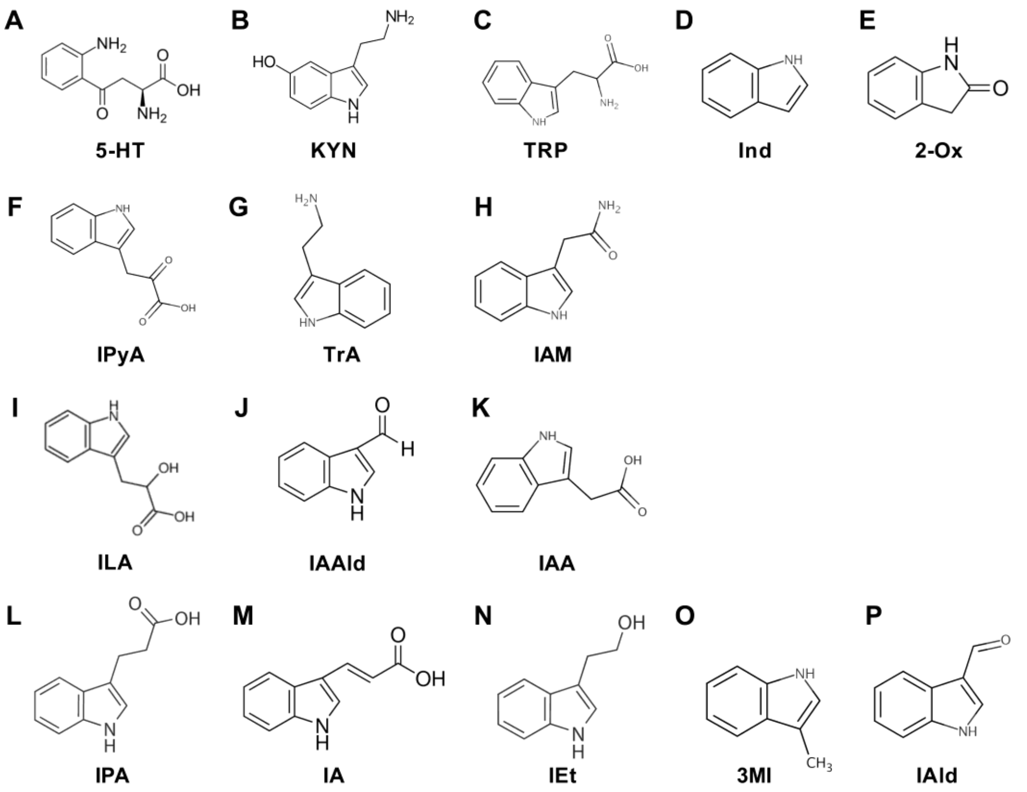

Fig. S6: Chemical structures of tryptophan (TRP) metabolites that involved in this study. List of compounds: serotonin (5-HT), kynurenine (KYN), tryptophan (TRP), indole (Ind), 2-oxindole (2-Ox), indole-3-pyruvic acid (IPyA), tryptamine (TrA), indole-3-acetamide (IAM), indole-3-lactic acid (ILA), indole-3-acetaldehyde (IAAld), indole-3-acetic acid (IAA), indole-3-propionic acid (IPA), indole-3-acrylic acid (IA), tryptophol (IEt), 3-skatole (3MI) and indole-3-carboxalolehyde (IAld).
