# Supplementary material for: Interplay between gut microbiota and tryptophan metabolism in type 2 diabetic mice treated with metformin
Source: Microbiol Spectr. 2024 Aug 20;12(10):e00291-24. doi: 10.1128/spectrum.00291-24 (PMC11448047; doi:10.1128/spectrum.00291-24)
Supplement: Table S1 — Preparation of standard solutions for standard curves. [file spectrum.00291-24-s0002.docx]

**Supplementary table**

**Supplementary Table S1** Preparation of standard solutions for standard curves

| Concentration/Compound  (μg/L) | 0.05μM | 0.1μM | 0.125μM | 0.25μM | 0.5μM | 1μM | 2.5μM | 5μM |
| --- | --- | --- | --- | --- | --- | --- | --- | --- |
| 5-HT | 8.8110 | 17.6220 | 22.0275 | 44.0550 | 88.1100 | 176.2200 | 440.5500 | 881.1000 |
| KYN | 10.4107 | 20.8214 | 26.0268 | 52.0535 | 104.1070 | 208.2140 | 520.5350 | 1041.0700 |
| TRP | 10.2114 | 20.4228 | 25.5285 | 51.0570 | 102.1139 | 204.2278 | 510.5695 | 1021.1390 |
| TrA | 8.0108 | 16.0216 | 20.0270 | 40.0540 | 80.1080 | 160.2160 | 400.5400 | 801.0800 |
| IAM | 8.7100 | 17.4200 | 21.7750 | 43.5500 | 87.1000 | 174.2000 | 435.5000 | 871.0000 |
| Ind | 5.8574 | 11.7148 | 14.6435 | 29.2870 | 58.5740 | 117.1480 | 292.8700 | 585.7400 |
| IPyA | 10.1595 | 20.3190 | 25.3988 | 50.7975 | 101.5950 | 203.1900 | 507.9750 | 1015.9500 |
| IAA-d2 | 8.8600 | 17.7200 | 22.1500 | 44.3000 | 88.6000 | 177.2000 | 443.0000 | 886.0000 |
| ILA | 10.2605 | 20.5210 | 25.6513 | 51.3025 | 102.6050 | 205.2100 | 513.0250 | 1026.0500 |
| IAAld | 7.9590 | 15.9180 | 19.8975 | 39.7950 | 79.5900 | 159.1800 | 397.9500 | 795.9000 |
| IAld | 7.2579 | 14.5158 | 18.1448 | 36.2895 | 72.5790 | 145.1580 | 362.8950 | 725.7900 |
| IAA | 8.7590 | 17.5180 | 21.8975 | 43.7950 | 87.5900 | 175.1800 | 437.9500 | 875.9000 |
| 2-Ox | 6.6574 | 13.3147 | 16.6434 | 33.2868 | 66.5735 | 133.1470 | 332.8675 | 665.7350 |
| IEt | 8.1597 | 16.3193 | 20.3991 | 40.7983 | 81.5965 | 163.1930 | 407.9825 | 815.9650 |
| IA | 9.3600 | 18.7200 | 23.4000 | 46.8000 | 93.6000 | 187.2000 | 468.0000 | 936.0000 |
| IPA | 9.4605 | 18.9210 | 23.6513 | 47.3025 | 94.6050 | 189.2100 | 473.0250 | 946.0500 |
| 3MI | 6.5590 | 13.1180 | 16.3975 | 32.7950 | 65.5900 | 131.1800 | 327.9500 | 655.9000 |
